# Inhibitory Effect of Methotrexate on Rheumatoid Arthritis Inflammation and Comprehensive Metabolomics Analysis Using Ultra-Performance Liquid Chromatography-Quadrupole Time of Flight-Mass Spectrometry (UPLC-Q/TOF-MS)

**DOI:** 10.3390/ijms19102894

**Published:** 2018-09-23

**Authors:** Zhiqiang Pang, Guoqiang Wang, Nan Ran, Hongqiang Lin, Ziyan Wang, Xuewa Guan, Yuze Yuan, Keyong Fang, Jinping Liu, Fang Wang

**Affiliations:** 1 Department of Pathogen Biology, College of Basic Medical Sciences, Jilin University, Changchun 130021, China; pangzq2812@mails.jlu.edu.cn (Z.P.); wanggq14@mails.jlu.edu.cn (G.W.); rannan17@mails.jlu.edu.cn (N.R.); wzy16@mails.jlu.edu.cn (Z.W.); guanxw15@mails.jlu.edu.cn (X.G.); yuanyz17@mails.jlu.edu.cn (Y.Y.); 2 Research Center of Natural Drug, School of Pharmaceutical Sciences, Jilin University, Changchun 130012, China; linhq17@mails.jlu.edu.cn (H.L.); liujp@jlu.edu.cn (J.L.); 3 Department of Pharmacology, College of Basic Medical Sciences, Jilin University, Changchun 130012, China; fangky15@mails.jlu.edu.cn

**Keywords:** methotrexate, rheumatoid arthritis, inflammation, metabolomics, lipid metabolism

## Abstract

Rheumatoid arthritis (RA) is a common autoimmune disease. The inflammation in joint tissue and system endanger the human health seriously. Methotrexate have exhibited a satisfactory therapeutic effect in clinical practice. The aim of this research was to establish the pharmacological mechanism of methotrexate on RA therapy. Collagen induced arthritic rats were used to identify how methotrexate alleviates inflammation in vivo. Lipopolysaccharide-induced inflammatory proliferation in macrophages was also be detected in vitro. The activation level of Nuclear factor kappa-light-chain-enhancer of activated B cells (NF-κB) and Nucleotide binding domain and leucine-rich repeat pyrin 3 domain (NLRP3)/Caspase-1 and related cytokines were examined by real-time PCR and western blotting or quantified with the enzyme-linked immunosorbent assay. Comprehensive metabolomics analysis was performed to identify the alteration of metabolites. Results showed that treating with methotrexate could alleviate the inflammatory condition, downregulate the activation of NF-κB and NLRP3/Caspase-1 inflammatory pathways and reduce the level of related cytokines. Docking interaction between methotrexate and caspase-1 was visualized as six H-bonds indicating a potential inhibitory effect. Metabolomics analysis reported three perturbed metabolic inflammation related pathways including arachidonic acid, linoleic acid and sphingolipid metabolism. These findings indicated that methotrexate could inhibit the onset of inflammation in joint tissue by suppressing the activation of NF-κB and NLRP3/Caspase-1 pathways and regulating the inflammation related metabolic networks.

## 1. Introduction

Rheumatoid arthritis (RA) is a chronic inflammatory joint disease that is characterized by synovial proliferation, joint destruction, and systemic inflammation [1,2]. It affects about 1–2% adult population globally [3]. RA begins with an autoimmune phase and is followed by an inflammation, which is mediated by the citrullinated antigen [4,5,6]. Despite the autoimmune reaction remains clinically silent, inflammation has the potential to cause severe pain and swelling [5].

The pathogenesis of RA may be associated with the involvement of environmental, infectious, and genetic factors [4]. Nuclear factor (NF)-κB activation had been demonstrated to be a crucial central role in the development and progression of RA because it not only participates in the production of many pro-inflammatory mediators but also regulates osteoclast genesis [7]. Besides, there also exists a high transcriptional and activated level of Nucleotide binding domain and leucine-rich repeat pyrin 3 domain (NLRP3) inflammasome in patients with RA [8]. The caspase-1 activation induced by NLRP3 inflammasome has been reported to be involved in RA [9]. Many inflammatory mediators contribute together to the pathogenesis of RA, despite the mechanism being still incompletely elucidated.

Metabolomics, as an emerging high-throughput technique for comprehensive analysis of endogenous small metabolites (<1 kDa) in biological system has been widely used to identify novel biomarkers and explore the potential pharmacological targets [10]. It usually focuses on the metabolic profile in vivo and directly shows us the metabolic changes that are associated with the pathological process of disease. Previous metabolomics studies have shown the potential of metabolic profiling to diagnose RA [11]. Recently, it has been applied extensively to investigate the pathogenesis of RA in patients and experimental arthritis models [11,12]. In addition, the pharmacological principles of many multi-targets medicine have also been clarified with non-targeted metabolomics techniques.

Conventional strategy of medical therapy for RA involves the Nonsteroidal anti-inflammatory drugs (NSAIDs), such as diclofenac [13]. But the side-effect and resistance of these pharmaceuticals usually cause a chronic pain and poor life quality and require a larger amount of resources for further management [14]. Methotrexate as an anti-folic acid metabolism medicine is widely used to inhibit the growth of carcinomas such as acute leukemia by blocking dihydrofolate reductase. As a result, humoral immunity is inhabited after the treatment [15]. Despite the patients with variant genotypes showed variable responsiveness towards methotrexate, it is still considered as a standard first-line medicine for RA in clinic practice and it has achieved a satisfactory therapeutic effect in over 60 percent RA cases [16]. However, the detailed mechanism behind this therapeutic effect has not been clarified completely yet.

In the present study, we established RA murine models and assessed the therapeutic effect of methotrexate in vivo and in vitro. At the same time, the inflammatory responses, mainly the activation of the typical NLRP3/Caspase-1 and NF-κB pathways in the whole joint tissue and macrophages were investigated. We tested the hypothesis that the treatment with methotrexate will alleviate the inflammatory condition by down-regulating the NF-κB and NLRP3/Caspase-1 pathways. Besides, the global view of metabolic profiles after the treatment of methotrexate was also identified. The molecular and protein interaction that is based on molecular dynamics virtual docking will contribute to the further pharmacological exploration.

## 2. Results

How methotrexate alleviate inflammation was studied in collagen induced arthritic (CIA) murine model and Lipopolysaccharide (LPS)-induced macrophages in present study.

### 2.1. Inhibitory Effect of Methotrexate on Murine CIA Paw Swelling

**Figure 1:**
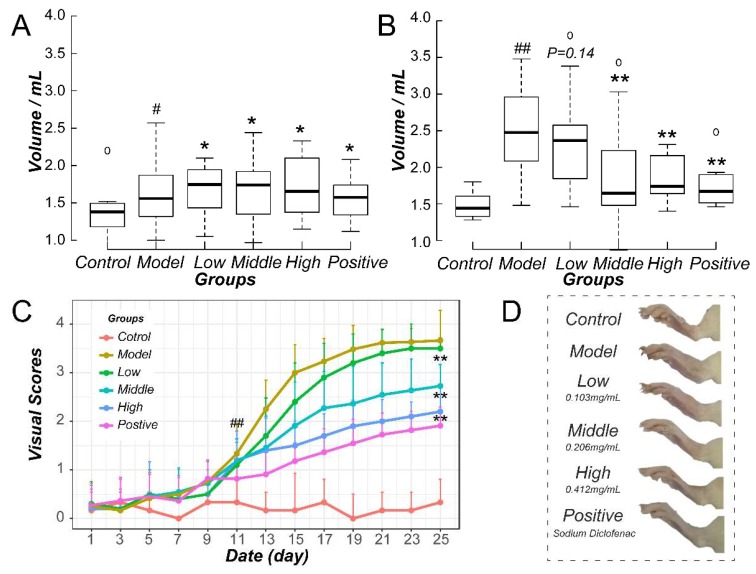
Evaluation on paw swelling of all groups. Swelling volume on Day 11 (**A**) and Day 25 (**B**). (**C**) The visual scores line of all groups including the whole time points. (**D**) The representative swelling states of all groups on Day 25. ^#^ *p* < 0.05 vs. Control. ^##^ *p* < 0.01 vs. Control. * *p* < 0.05 vs. Model. ** *p* < 0.01 vs. Model. The free small circles in (**B**) represent the mild outlier samples.

Swelling of CIA paws showed a statistical significance at 11th day according to the visual scores after the booster injection. Paw swelling volume was examined for all experimental animals then. As shown in Figure 1A, the experimental arthritis groups that were injected with collagen emulsion showed a significant increase in paw swelling volume when compared with the control (Control) group (*p* < 0.05). Drugs administration began at day 11 and continued until the visual scores of the CIA model (Model) group came to a platform stage. As expected, the endpoint of the administration came at day 25. The low dosage (Low) group showed a trend of inhibitory effect on paw swelling when compared with the model group (*p* = 0.14), as shown in Figure 1B,C. However, middle dosage (Middle), high dosage (High), and positive drug diclofenac sodium (DCS, Positive) group showed a significantly reduced swelling volume and visual scores, as indicted by Figure 1B–D.

### 2.2. Downregulation of Methotrexate on NF-κB/NLRP3 Pathways in Murine Model

**Figure 2:**
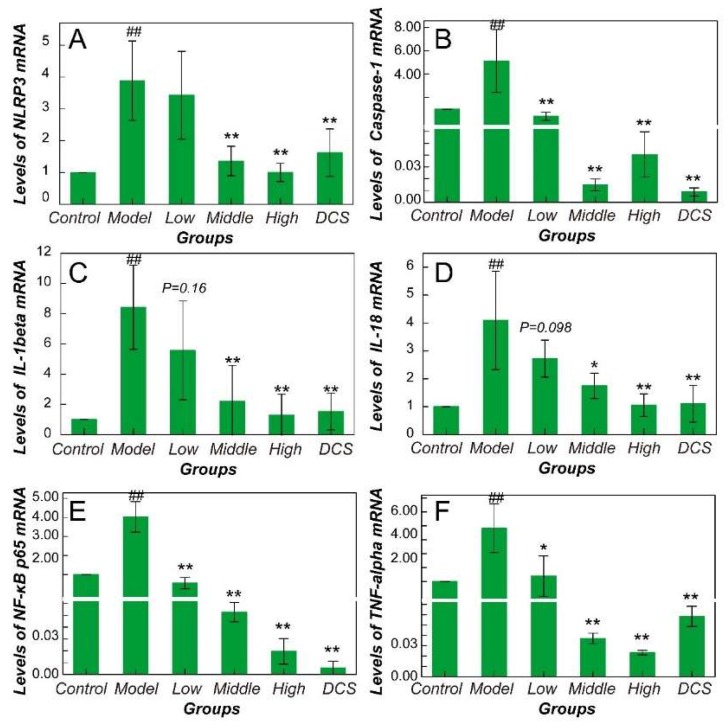
Quantification results of real-time PCR in all murine groups. Gene expression level of (**A**) NLRP3, (**B**) caspase-1, (**C**) IL-1β, (**D**) IL-18, (**E**) NF-κB p65, (**F**) TNF-α. All values were normalized against β-actin. DCS, Diclofenac sodium. ^##^ *p* < 0.01 vs. Control. * *p* < 0.05 vs. Model. ** *p* < 0.01 vs. Model.

NF-κB and NLRP3/Caspase-1 pathways mRNA expression was remarkably higher in CIA ankle joint tissue in Model compared with Control indicating a significant activation (Figure 2). In detail, NF-κB p65 and TNF-α gene expression in all the drug administrated groups decreased significantly (*p* < 0.05). Level of NLRP3 gene expression in Middle and High group is different from Model group significantly (*p* < 0.01). The mRNA level of Caspase-1 in drug-administrated groups showed an obvious decrease compared with the model animals (*p* < 0.01) in a dose-independent manner. However, IL-1β and IL-18 gene expression only decreased significantly in Middle, High, and Positive groups (*p* < 0.01). The IL-1β and IL-18 gene expression in the Low group simply showed a trend of decrease.

**Figure 3:**
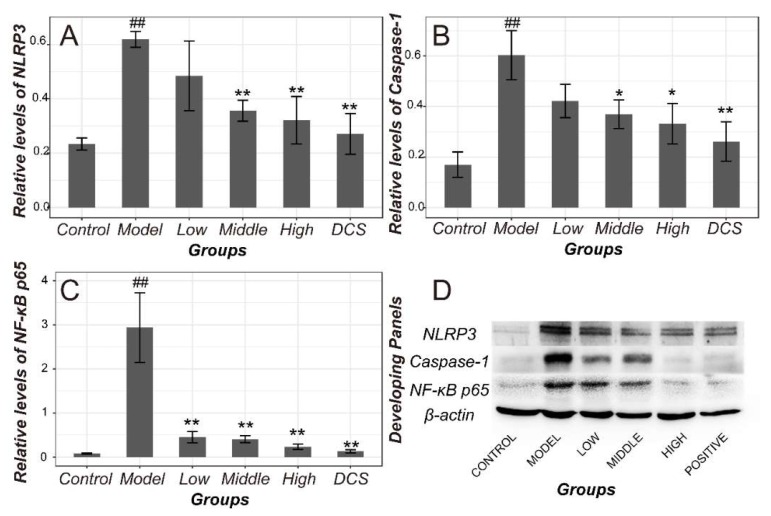
Protein levels of NF-κB and NLRP3/Caspase-1 pathway in CIA rats. Results of optical density of NLRP3 (**A**), caspase-1 (**B**), NF-κB p65 (**C**) estimated by integrated optical density (IOD index) and normalized against β-actin in all groups. (**D**)The representative Chemiluminescencez panels of all protein membranes. DCS, Diclofenac sodium. ^##^ *p* < 0.01 vs. Control. * *p* < 0.05 vs. Model. ** *p* < 0.01 vs. Model.

NLRP3 protein in Model rats increased significantly (*p* < 0.01). So, as the protein expression level of caspase-1 and NF-κB p65 in Model (*p* < 0.05), as displayed in Figure 3. All of the drug administrated groups showed a downregulated expression of NLRP3, caspase-1, and NF-κB p65 protein when compared with Model. Especially, they were downregulated remarkably in Middle, High and Positive group compared with Model (*p* < 0.05 or *p* < 0.01). Moreover, NF-κB p65 protein in Low group also showed a statistical significance (*p* < 0.05).

**Figure 4:**
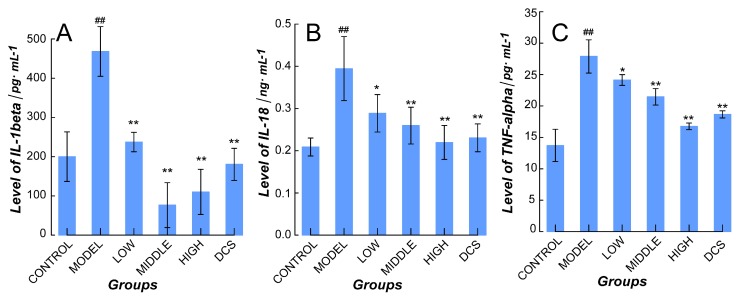
Quantification of plasma cytokines related with NF-κB and NLRP3/Caspase-1 pathways in all CIA murine groups. (**A**) IL-1β, (**B**) IL-18, (**C**) TNF-α levels in all groups. DCS, Diclofenac sodium. ^##^ *p* < 0.01 vs. Control. * *p* < 0.05 vs. Model. ** *p* < 0.01 vs. Model.

Besides, the quantification assay results of IL-1β, IL-18, and TNF-α in plasma were represented in Figure 4. The NF-κB and NLRP3/Caspase-1 pathways related cytokines in plasma of Model increased significantly (*p* < 0.01 vs. Control) and decreased in all the drug-administrated groups (*p* < 0.05 for Low vs. Model and *p* < 0.01 for Middle, High, and Positive groups vs. Model, respectively).

### 2.3. Methotrexate Suppressed the Inflammatory Proliferation of Macrophages

**Figure 5:**
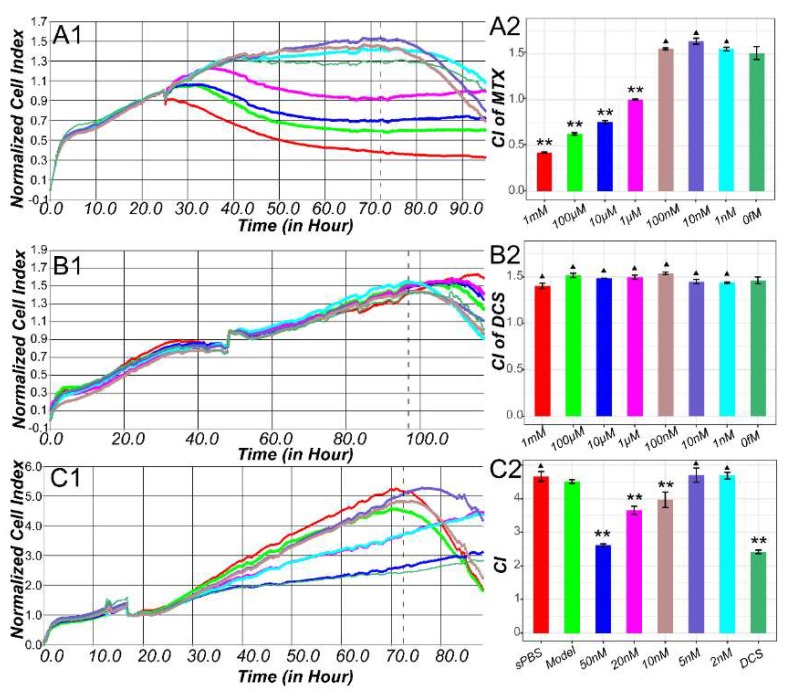
Proliferation curves of RAW 264.7 monitored with RTCA system and the statistic results. Cytotoxic assay of methotrexate (**A**) and Diclofenac sodium (DCS) (**B**) with a series of geometric concentrations from 1 nM to 1 mM. The treatment of methotrexate was performed at 24th h. (**A1**) The proliferation curve of methotrexate with different dosages. (**A2**) Statistics of cell indexes in **A1** at 72nd h The treatment of DCS was performed at 48th h. (**B1**) The proliferation curve of DCS with different dosages. (**B2**) Statistics of cell indexes in **B1** at 96th h. (**C1**) Anti-inflammation curves of methotrexate with different dosages and DCS (1 nM). All cellular groups were treated with LPS at 12th h for 4 h. Cellular Control (Stimulated with LPS followed by PBS, sPBS) was free of LPS after the 4 h’ stimulation. The cell indexes were statistically evaluated at 72nd h. ** *p* < 0.01 vs. Model. ^▲^ *p* > 0.05 vs. 0 fM in **A2** and **B2**, vs. Model in **C2**.

The cytotoxicity of both methotrexate and DCS on RAW 264.7 was monitored with Real Time Cellular Analysis (RTCA) system and displayed as Figure 5. After a continuous incubation with different drug concentration, the cytotoxicity was evaluated at 72 h for methotrexate and at 96 h for DCS in case of proliferation recession. As shown in Figure 5A1,A2, methotrexate significantly inhibited the cell proliferation over the range of 1 μM to 1 mM indicating a cytotoxic effect (*p* < 0.01). DCS did not exhibit any suppression on the proliferation with all drug concentrations (Figure 5B, *p* > 0.05). The non-cytotoxic concentrations were set as 50, 20, 10, 5, 2 nM for methotrexate and 1 mM for DCS to test their anti-inflammatory effects. As a result, compared to the Model, the Control cells exhibited a similar inflammatory proliferative status (*p* > 0.05). DCS and methotrexate in 50, 20 and 10 nM significantly reduced the inflammatory proliferation (*p* < 0.01). However, there did not exist any difference on proliferative characteristics compared to the Model after treating with methotrexate of 5 and 2 nM (*p* > 0.05).

### 2.4. Methotrexate Inhibited NF-κB and NLRP3/Caspase-1 Pathways in Macrophages

**Figure 6:**
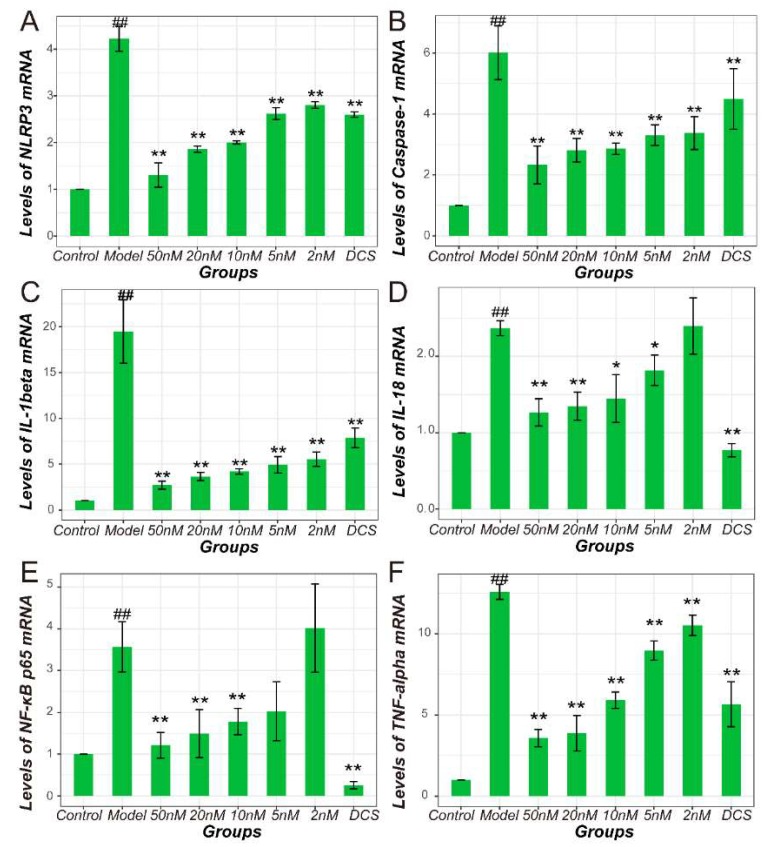
Quantification results of real-time PCR in all cellular groups. Gene expression level of (**A**) NLRP3, (**B**) caspase-1, (**C**) IL-1β, (**D**) IL-18, (**E**) NF-κB p65, (**F**) TNF-α. All values were normalized against β-actin. DCS, Diclofenac sodium. ^##^ *p* < 0.01 vs. Control. * *p* < 0.05 vs. Model. ** *p* < 0.01 vs. Model.

The mRNA expression level of all related genes in RAW 264.7 inflammatory cellular model after the treatment was shown in Figure 6. All genes including were remarkably upregulated in inflammatory cellular models (*p* < 0.01). After the treatment with methotrexate, the expression of all investigated genes was significantly inhibited in dose-dependent manner (*p* < 0.01 vs. Model). However, methotrexate with 2 and 5 nM only displayed a potential to downregulate the expression of NF-κB p65 and IL-18 genes (*p* > 0.05 vs. Model).

**Figure 7:**
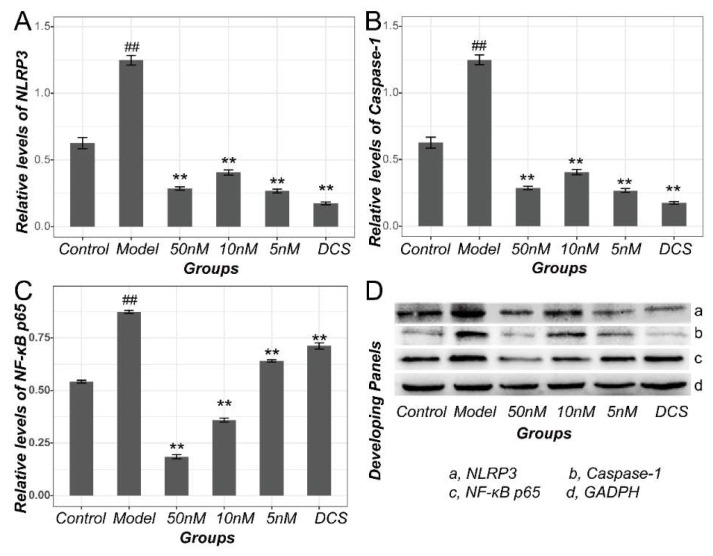
Protein levels of NF-κB and NLRP3/Caspase-1 pathway in RAW 264.7 cells. Results of optical density of NLRP3 (**A**), caspase-1 (**B**), NF-κB p65 (**C**) estimated by integrated optical density (IOD index) and normalized against β-actin. (**D**)The representative Chemiluminescencez panels of all protein membranes. DCS, Diclofenac sodium. ^##^ *p* < 0.01 vs. Control. ** *p* < 0.01 vs. Model.

**Figure 8:**
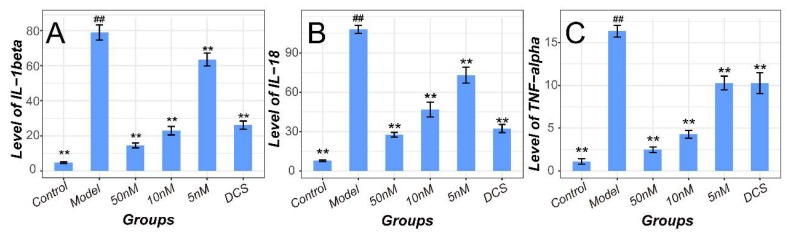
Quantification of supernatants cytokines related with NF-κB and NLRP3/Caspase-1 pathways in all cellular groups at 48th h (**A**) IL-1β, (**B**) IL-18, (**C**) TNF-α levels in all groups. DCS, Diclofenac sodium. ^##^ *p* < 0.01 vs. Control. ** *p* < 0.01 vs. Model.

Consistent with the results in CIA murine model, proteins including NLRP3, Caspase-1 and NF-κB p65 in inflammation cellular Model rose sharply compared with Control (*p* < 0.01). Their levels decreased significantly after the therapy with methotrexate and DCS in a dose-independent manner for NLRP3 and Caspase-1 or in a dose-dependent manner for NF-κB p65 (*p* < 0.01 vs. Model) as shown in Figure 7. However, the pathways related cytokines including IL-1β, IL-18 and TNF-α increased significantly in LPS induced cellular inflammation Model (*p* < 0.01 vs. Control) and decreased in a dose-dependent manner (*p* < 0.01 vs. Model, Figure 8).

### 2.5. Multivariate Analysis and Differentiated Metabolic Patterns

Metabolomics analysis on the metabolic perturbation after the treatment was performed with Ultra-Performance Liquid Chromatography-Quadrupole Time of Flight-Mass Spectrometry (UPLC-Q/TOF-MS) systems. The stability and suitability of spectrum and chromatogram was evaluated based on the 10 ions in both ESI^+^ and ESI^−^ modes from six quality control (QC) sample injections respectively. All relative standard deviation (RSD) values including repeatability and intermediate precision were calculated as less than 6.0% (Table S1), which indicated a good confidence for subsequent analysis.

**Figure 9:**
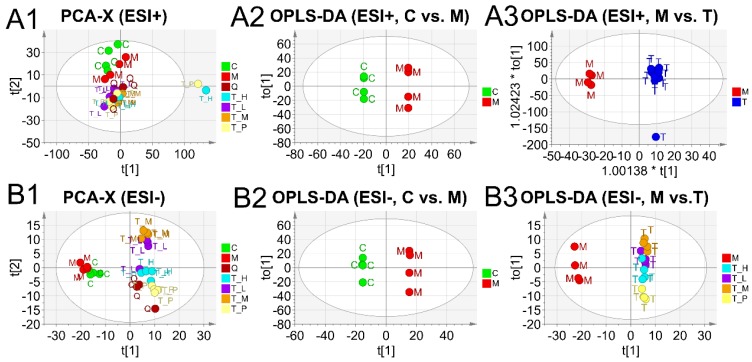
Multivariate analysis results of Ultra-Performance Liquid Chromatography-Mass Spectrometry (UPLC-MS). Principle Component Analysis (PCA) and orthogonal projections to latent structures discriminant analysis (OPLS-DA) models were displayed as two-dimensional plots. PCA of all samples in electrospray ionization in positive (ESI+) mode (**A1**) and in electrospray ionization in negative (ESI−) mode (**B1**) (PC1 = 51.1%, PC2 = 8.25% for ESI+ and PC1 = 28%, PC2 = 11.6% for ESI−). The OPLS-DA discrimination score plots between different groups: (**A2**) Control vs. Model in ESI+, *p* < 0.00001; (**A3**) Model vs. Treatment groups in ESI+, *p* = 0.015; (**B2**) Control vs. Model in ESI−, *p* < 0.00001; (**B3**) Model vs. Treatment groups in ESI−, *p* < 0.0001. Control was abbreviated as C, Model as M, Treatment with low, middle, high dosage as T_L, T_M and T_H respectively. Treatment with positive DCS as T_P, QC as Q. In PCA, *t* [1] are the first variables summarized from the original data and *t* [2] are the second ones. In OPLS-DA, *t* [1] is the predictive X-scores and to [1] is the Orthogonal X-scores. All *p* values were estimated with *CV-ANOVA* test. * in A3 means the multiple magnification to the coordinate axes.

Principle component analysis (PCA) as an unsupervised lowering-dimension pattern recognition model revealed the inherent similarity among the different samples in ESI+ and ESI− modes visually (Figure 9A1,B1). Sample from different groups displayed as intrinsic clusters indicating a lager similarity within them under both ESI+ (R2 = 64.05%, Q2 = 47.80%) and ESI− (R2 = 54.68%, Q2 = 33.90%) modes.

4 orthogonal projections to latent structures discriminant analysis (OPLS-DA) models were also established with a supervise based on the experimental treatment groups. As shown in OPLS-DA plots of Figure 9, all samples in different models were appreciably separated from each other indicating there exists no extremely abnormal sample. As a result, OPLS-DA models established can be used to identify the differentiated metabolites and potential biomarkers (R2 = 99.99%, Q2 = 78.32% for C vs. M in ESI+, R2 = 99.32%, Q2 = 82.46% for M vs. T in ESI+; R2 = 99.99%, Q2 = 45.02% for C vs. M in ESI−, R2 = 99.56%, Q2 = 97.10% for M vs. T in ESI−). Besides, the validation of all OPLS-DA models for differentiated metabolites analysis have been confirmed by cross validation ANONA analysis (*p* < 0.0001).

### 2.6. Global Profiles of Distinct Metabolites and Perturbed Metabolic Pathways

Distinct metabolites were selected according to the Variable Importance for the Projection (VIP) indexes that were calculated based on the OPLS-DA models above. Metabolites with VIP values more than 1.0 and *p* values were less than 0.05 were chosen for tandem mass spectrometry (MS/MS) tandem identification and metabolic pathway analysis. A total of 24 remarkably changed metabolites in plasma were determined with the details listed in Table 1. Comparison details were provided in Figures S1–S24.

**Figure 10:**
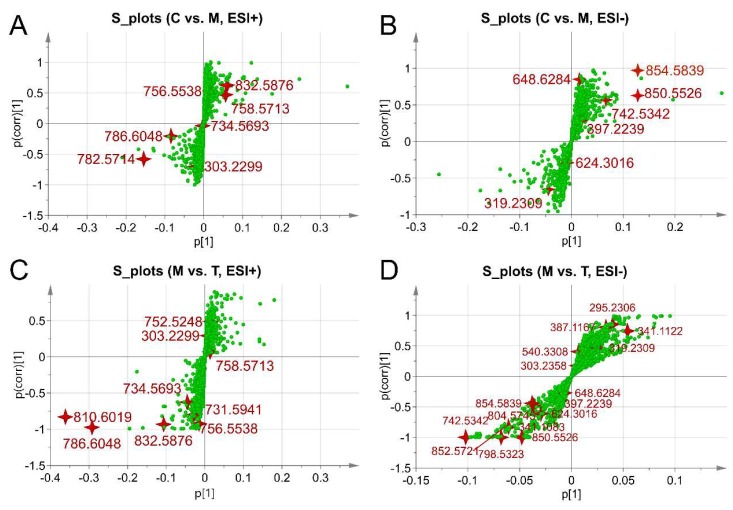
S-plots of all differentiated metabolites based on the OPLS-DA models. C vs. M in ESI+ (**A**) and in ESI− (**B**); M vs. T in ESI+ (**C**) and in ESI− (**D**). All of the significantly changed metabolites identified were marked as red four-polar stars. Other components were green points. The *p* [1]-axis describes the magnitude of each variable in X. The *p*(corr) [1]-axis represents the reliability of each variable in X. Control was abbreviated as C, Model as M, Treatment with drugs as T.

All of the identified metabolites ions were also displayed in S-plots (Figure 10), which combined correlation and covariation values of the OPLS-DA models. The edging distribution of all the distinct metabolites in the loading plot revealed enough significance and confidence to illustrate the group difference within the OPLS-DA models. All of the potential biomarkers were evaluated with receiver operating characteristic curve (ROC), as shown in Figure S25. Metabolites with AUC > 0.9 were defined as high diagnostic accuracy. As a result, 19 differentiated metabolites were identified as potential biomarkers with high sensitivity and specialty.

**Figure 11:**
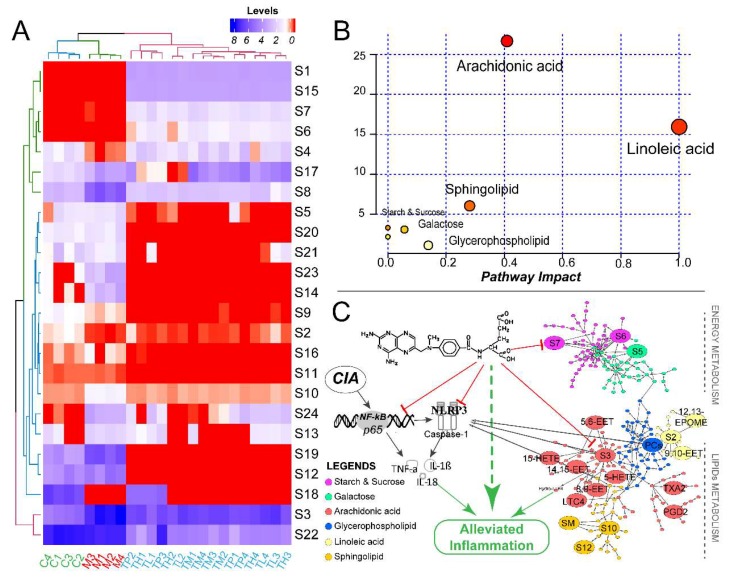
Differentiated metabolites and perturbed metabolic pathways. (**A**) The heatmap all of differentiated metabolites. (**B**) Results of metabolic pathways produced by MetaboAnalyst 4.0. The pathway Impact value described the significance of the metabolic pathways. (**C**) The comprehensive analysis about the invention from methotrexate and interaction between inflammatory pathways (including NF-κB and NLRP3/Caspase-1) and metabolic pathways (arachidonic acid, linoleic acid, sphingolipid, glycerophospholipid, Galactose, and starch and sucrose metabolic pathways). EET, epoxyoctadecenoic acid; HETE, hydroxyeicosatetraenoic acid; LTC4, Leukotriene C4; SM, Sm(d18:0/18:1); TXA2, Thromboxane A2. PGD2, Prostaglandin D2.

Besides, the relative levels of the distinct metabolites were displayed with a heatmap after the mathematic LOG transformation (Figure 11A). Samples were naturally clustered into three major branches, which is consistent with the biological groups. It is noted that the drug-administrated samples share a large similarity on their metabolic patterns.

Eight metabolic pathways were identified to be involved (Table S3, Figure 11B) according the results from MetaboAnalyst 4.0 platform. Three of them, including arachidonic acid, linoleic acid, and sphingolipid metabolisms were significantly perturbed (Impacts > 0.1 and −log(P) > 5). The comprehensive interaction between inflammatory pathways and metabolic pathways were summarized and displayed in Figure 10C.

### 2.7. Potential Docking Mode of Methotrexate within Caspase-1 Protein

**Figure 12:**
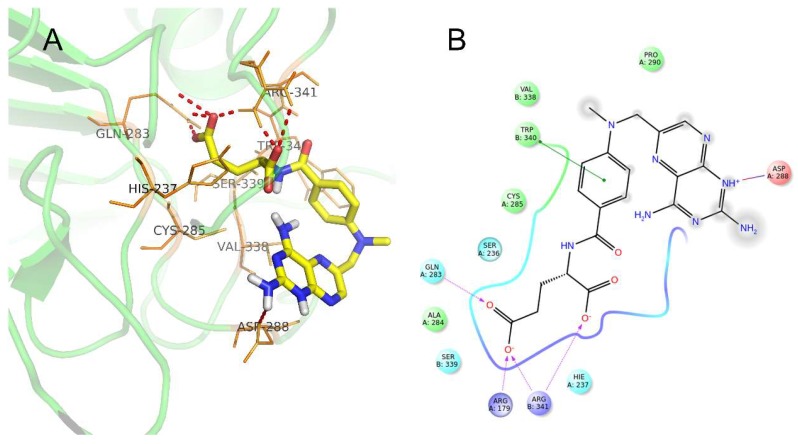
Docking interaction modes of methotrexate within the binding pockets of caspase-1. (**A**) The 3-dimensional polar contacts between methotrexate and caspase-1. The light green cartoon is caspase-1 prepared by pymol. The red dotted line is the polar contacts. The thin orange sticks are the interactive residues of caspase-1. The thick sticks are the methotrexate (Yellow are carbon atoms, Blue are nitrogen atoms, Red are oxygen atoms, White are some of the hydrogen atoms). (**B**) two-dimensional display of the interaction between the ligand and the protein (The colorful balls at the edge are related amino acids. Polar contacts are displayed with different lines. Purple dotted lines represent the polar contacts with Oxygen anion, green line represents with Benzene, blue red line represents with ammonium).

Molecular docking performed to investigate the potential interaction between methotrexate and the critical inflammatory cytokines catalyzation related enzyme, caspase-1 revealed an accurate docking site and the appropriate conformation. The precise modes of interaction and relative binding energy were measured as Table 2. Moreover, the number of H-bonds and the docking scores was calculated with the Schrödinger demo. As shown in Figure 12A,B, both the molecular conformation of methotrexate and the polar contacts (seven contacts, including six H-bonds and one other polar contacts) were displayed. The residue ARG-341 has the most contacts with the ligand.

## 3. Discussion

Collagen-induced arthritis (CIA) in rats is the most widely used model for RA because it shares many similar immunological and pathological features with RA in patients [17]. Innate immune components, especially macrophage is a prime source of pro-inflammatory cytokines and critically contribute to RA pathology [3,18]. In present study, we established the CIA murine model and the inflammatory macrophage cellular model for pharmacological research on the anti-rheumatic effect of methotrexate. We discovered that methotrexate could alleviate inflammatory swelling in CIA rats and inhibit the inflammatory proliferation of macrophages by suppressing NF-κB and NLRP3/Caspase-1 pathway and downregulate the level of pathway related cytokines. Besides, methotrexate has the potential to inhibit catalytic activity of caspase-1 directly. Three inflammation related metabolic pathways including arachidonic acid, linoleic acid and sphingolipid metabolism also participated in the pathogenesis of RA and were regulated significantly after the treatment with methotrexate.

NF-κB is responsible for the regulation of inflammation and immune responses and it is involved in the pathogenesis of RA. The damage-associated molecular patterns binding to Toll-like receptors, as the priming signal activate the upregulation of the NF-κB nucleus transfer [3,19]. The translocated free NF-κB then activates its target genes to transcribe various kinds of inflammatory mediators, such as proIL-1β, proIL-18, and NLRP3 [19,20]. NLRP3 protein was constructed as NLRP3 inflammasome assembly with other components including caspase recruitment domain containing protein 8, pro-caspase-1 and so on together. The activation of caspase1, which is mediated by the inflammasome further induces maturation of IL-1β and IL-18 in RA [21]. The similar activation of NF-κB and NLRP3/Caspase-1 pathways were also observed in CIA ankle joint tissues and LPS-induced macrophages in present study. A great deal of evidence has been accumulated and revealed the beneficial effect of direct inhibition of NF-κB on inflammatory disease [22,23]. Herein, methotrexate inhibited the activation of NF-κB and NLRP3/Caspase-1 pathways in joint tissue and decreased the level of systematic cytokines in a dose dependent manner. The activity-inhibiting binding of methotrexate within caspase-1 may have the potential to partly explain the therapeutic effect, but the detailed mechanism is not clear.

Metabolomics techniques as a high-throughput approach have been extensively utilized to investigate the pathogenesis of RA and therapeutic targets of anti-RA drugs [11,24]. In the present study, we discovered that arachidonic acid, linoleic acid, and sphingolipid metabolism perturbed and they were significantly regulated after administrating with methotrexate.

Arachidonic acid, a component that is released from cellular membrane would be metabolized as a variety of metabolites, including EETs, HETEs, prostaglandins, thromboxane, and leukotrienes by cyclooxygenases or lipoxygenases and promote the nuclear translocation of NF-κB, which is at least partly induced by eicosanoids [25,26]. At the same time, the NF-κB pathway is the most critical activator for NLRP3 inflammasome and the massive production of lipid mediators [27]. EETs are a series of lipid mediators with critical physiological functions that include anti-inflammation, anti-hypertension, and organ protection effects [28]. The anti-inflammation effect is associated with the regulation of NF-κB translocation, as described previously [29]. Methotrexate also increased the production of EETs and inhibited the activation of NF-κB in present study. Prostaglandins and leukotrienes, which was believed to cause pain and activate the formation of cytokine were reduced after the treatment of methotrexate. So did the level of thromboxane A2. HETEs as a class of lipids with distinct inflammation bioactive roles were also been influenced by methotrexate. In brief, methotrexate modulate the NF-κB and NLRP3/caspase-1 inflammasome-mediated arachidonic acid metabolic lipid profiles, inhibit the release of pro-inflammatory lipids and promote the production of anti-inflammatory mediators in CIA murine rats.

The precursor of arachidonic acid, linoleic acid can be converted to arachidonic acid and epoxides of linoleic acid (including 9,10-EpOME and 12,13-EpOME) [30]. The EpOMEs that are produced by neutrophils and macrophages are considered as leukotoxins and they contribute to the pathogenesis of inflammatory disease, such as acute respiratory distress syndrome [31]. We observed a significant increase in CIA rats and a decrease of EpOMEs after the treatment of methotrexate. Linoleic acid itself seems to have potent anti-inflammatory effect, which is associated with the diminished NF-κB binding activity and delayed translocation of NF-κB [32,33]. A lower level of linoleic acid reported before in RA serum is consistent with our results in CIA [34]. Linoleic acid also has the potential to ameliorate the high expression level of NLRP3 genes and reduced the release of mature IL-1β and other cytokines [35,36]. Despite that methotrexate could damage the dietary absorption of linoleic acid [37], it had already been reported to increase linoleic acid in carcinoma cell and was demonstrated in present study in CIA rats to play its anti-inflammation role [38].

Sphingolipids as a class of bioactive lipids, including ceramides, sphingomyelins, and lactosylceramides play a key modulating role in cell cycle, apoptosis, and inflammatory responses [39]. They could also participate in the osteoblast crosstalk on bone homeostasis [40]. Sphingomyelin, a critical structural component of biological membranes is tightly associated with the activation of NF-κB [39,41]. The hydrolyzed metabolites of sphingomyelin, ceramides have already been linked to the inflammation process and act as a mediator inducing the activation of NF-κB and the sensitivity of NLRP3 inflammasome [42]. Another pro-inflammatory key branching point of sphingolipid biosynthesis, lactosylceramides were also reported to involve the activation of Ras/NF-κB and inflammatory gene expression [43]. Similar to our results, the level of these sphingolipids increased significantly in RA synovial fluid [44]. Therapeutic effect of methotrexate on CIA murine sphingolipid metabolism was reported for the first time in the present study. We demonstrated that methotrexate might have downregulated the NF-κB and NLRP3/caspase-1 pathways at least partly by intervening the sphingolipid metabolism.

RA was accompanied with a differentiated energy metabolism and expenditure [45]. Trehalose was reported to inhibit the inflammation by regulating NF-κB [46]. Herein, we observed a mild alteration of energy metabolism in CIA rats after the administration of methotrexate, indicating a potent mechanism that is unestablished. Glycerophospholipids are a critical series compounds for participating in many biological regulatory processes, including the pathogenesis of RA [47]. In present study, we identified large series phosphatidylcholines, most of which had long polyunsaturated side chains. The detailed role of every phosphatidylcholines metabolites was unknown, but their potential predictive effects for diagnosis and therapy will benefit for the development and investigation of biomarkers in clinical practice.

Although the subjects with RA mainly manifests a pathological change in synovial joint and cartilage, recent studies reported that the autoimmune response in patients with RA was initiated outside the joint, affected different parts of the body, and resulted in multi-organ disorders inevitably [48]. The inflammation related pathways and cytokines that we detected in the joint ankle tissue could reflect the pathophysiological changes more sufficiently. Given that many immune components such as innate B lymphocyte and adaptive T lymphocyte have been reported to participate the pathogenesis of RA [49], whether methotrexate could potentially regulate the inflammatory mechanism of these cells need further elucidation. Untargeted metabolomics in present study was utilized to analyze all the measurable molecules in a sample, including chemical unknowns. But, the extremely limited ability to quantify with the untargeted metabolomics require us to perform the targeted lipidomic research on the RA patients treated with methotrexate. Small size for metabolomics study is the other limitation of this study. Besides, as a classical chemical therapeutic drug, the unknown side effects after inhibiting the potential target also need our further evaluation. Nevertheless, this pilot study will be a pioneer for further determination on the discovered metabolism pathways.

## 4. Materials and Methods

### 4.1. Experimental Animal and Ethical Approval

Female Wistar rats, aged six to eight weeks and weighing 160–180 g were provided by Yi-Si Animal Corporation (Changchun, China, Certificate No. SCXK (JI)-2016-0003). All of the rats were housed individually in a 12 h’ light and dark circular room. The environment of the room was under control (temperature 23 ± 2 °C and humidity 40–60%). The animals could obtain food and water ad libitum. All the animal experiments protocols were conducted based on the guidelines for the administration of laboratory animals (Directive 86/609/EEC in the Protection of Animals Used for Experimental and Other Scientific Purposes, 1986) and were approved by the Institutional Animal Care and Use Committee of Jilin University (No. SCXK-2013-0001, 27th May 2017).

### 4.2. RA induction and Drug Administration

Animals were randomly divided into six groups: Control Group (*n* = 12), Model Group (*n* = 12), Low Concentration Group (*n* = 12), Middle Concentration Group (*n* = 12), High Concentration Group (*n* = 12), and Positive Medicine Group (*n* = 12). After adaptive feeding for five days, fresh emulsion was prepared by emulsifying the Incomplete Freund’s Adjuvant (Chondrex, Inc.2607 151st Place NE Redmond, WA 98052, USA. Cat. 7002) with the bovine collagen solution (Chondrex, Cat. 20022) in the ice water. The induction of CIA was performed according to the manufacturer’s instructions. Briefly, the animals were induced twice at 0th and 7th day, respectively. After the booster injection, animals were administrated intragastrically with methotrexate (Tong Hua Mao Xiang Pharmaceutical Co., Ltd., Tong Hua City, Jilin Province, China, Approval Number: H22022674, 15th July 2011) twice a week (0.103 mg/mL for Low, 0.206 mg/mL for Middle, 0.412 mg/mL for High, 5 mL/kg Body weight) or diclofenac sodium (Warrant Pharmaceutical, Hunan Province, China, Approval Number: H20067776, 23rd March 2016) everyday (2.08 mg/mL for Positive, 5 mL/kg Body weight) once the swelling of paw appeared in the Model. Model and Control were administrated with distilled water.

### 4.3. Assessment on RA Swelling

The experimenter recorded the visual scores according to the recommended scoring system from manufacturer of Chondrex every two days. Paw swelling volume was also recorded before and after drug administration with paw volume measuring instrument (PV-200, Chengdu Tai Meng Software Co., Ltd., Chengdu City, Sichuan Province, China). The end-line of the paw volume measurement was defined as 0.3 cm above the ankle. Experimenter continued to score the swelling level until the animals were sacrificed. The observer was blind to the biological groups of experimental animals.

### 4.4. Sacrifice and Sample Collection

All of the animals were sacrificed when the average visual score of Model measured nearly and stably up to 4. The animals were anesthetized with sodium pentobarbital and sacrificed by collecting whole blood from abdominal aorta directly into the heparin sodium treated vacuum tube. Plasma samples was prepared by centrifuging the blood at 3000 rpm for 15 min at 4 °C and stored at −80 °C. The preparation of ankle joints tissue was performed as described previously [50]. All of the ankle joints tissues were stored at liquid nitrogen for RNA and protein extraction.

### 4.5. Cell Viability Assay with Real Time Cellular Analysis

Proliferation of RAW 264.7 cells, as obtained from American Type Culture Collection (ATCC TIB-71) was monitored with Real Time Cellular Analysis system (xCELLigence RTCA S16, ACEA Biosciences, San Diego, USA) in Dulbecco’s modified Eagle’s medium (DMEM, Caisson, Smithfield, UT, USA) containing 10% fetal bovine serum (FBS, Genedirex, Las Vegas, NV, USA) with 1% penicillin/streptomycin in a humidified atmosphere with 5% CO_2_ in a 37 °C incubator. The toxicity assays of both methotrexate and diclofenac sodium were performed with RTCA system at a series of geometric concentrations (1 mM, 100 μM, 10 μM, 1 μM, 100 nM, 10 nM, 1 nM, 0 fM). The non-toxic dosages of methotrexate and diclofenac sodium were selected to investigate their anti-inflammation effect. 3500 cells in every well were cultured in the E-plates of the RTCA system and stimulated with lipopolysaccharide (LPS, 1 μg/mL, Sigma, Darmstadt, Germany. Cat. L2630) at 12th h for 4 h. After stimulating with LPS, the medium of Model group was continued to be under the stimulation of LPS. The anti-inflammation groups were treated with methotrexate in different concentrations or diclofenac sodium respectively. Control was cultured with 10% DMEM+PBS after the four hours’ stimulation with LPS.

### 4.6. Cell Culture and Induction of Inflammation

RAW 264.7 cells were seeded in the six-well plates with 1 × 10^5^ cell/well and cultured in the medium described above. The stimulation and treatment steps are consistent with the RTCA. All cells were harvested at the endpoint which was determined according to the highest peak of RTCA monitor for RNA and protein extraction. The supernatants were collected after removing cell debris by centrifugation prior to use for enzyme-linked immunosorbent assay (ELISA).

### 4.7. qPCR Assay

About 100–150 mg ankle tissues or 2 × 10^6^ cells were homogenized in 1.0 mL or 200 μL TRIzol reagent (Invitrogen, Waltham, MA, USA. Cat. 15596-026). The dissected tissue was homogenized with magnetic beads homogenizer (Gering Scientific Instruments (Beijing) Ltd., Beijing, China) at 4 °C. Total RNA was extracted to be reversely transcribed and amplified according to the manufactural instructions (RevertAcid First Strand cDNA Synthesis Kit, Thermo Scientific, Waltham, MA, USA. Cat. 1662). All of the primers are listed in Table S2. Gene expression level of NLRP3, Caspase-1, IL-1β, IL-18, TNF-α, and NF-κB p65, β-actin was analyzed after 40 cycles while using Applied Biosystems StepOne Real-Time PCR System. The qPCR amplification mix was prepared as the recommended protocol of FastStart Universal SYBR Green Master (ROX, Roche, Basel, Switzerland. Cat. 04913914001,). The expressive level was calculated with the 2^−ΔΔCt^ method.

### 4.8. Western Blotting and ELISA Assay

About 100–150 mg arthritic ankle joint tissue or 2 × 10^6^ cells were homogenized as described above in 1.0 mL or 150 μL reagent of tissue protein lysate (RIPA: PMSF = 100:1) for protein extraction. The concentration of protein was determined with BCA kit (Beyotime Biotechnology Shanghai City, China, Cat. P0010). Briefly, all of the denatured protein samples were subjected by 12% SDS-PAGE gel and transferred to a polyvinylidene difluoride (PVDF) membrane (Millipore, Biosharp, Hefei City, China.) followed by blocking for 1 h and incubating with antibodies against NLRP3 (abcam, Cambridge, USA, Cat. ab214185), Caspase-1 (abcam, ab54932), NF-κB p65 (abcam, Cat. ab16502), and β-actin (Bioss, Beijing City, China. Cat.bs-0061R), respectively, overnight at 4 °C. After being incubated with secondary antibodies (Beyotime, Cat. A0208 and Cat. A0216) labeled with horseradish peroxidase (HRP), all the membranes were detected with the chemiluminescence (ECL) system and analyzed with Image-Pro Plus 6.0 software.

The plasma and cell supernatants were thawed on ice for the quantification of inflammatory cytokines. IL-1β, IL-18, TNF-α were determined by ELISA (Muti Sciences, Hangzhou City, China, Cat. EK301B5/2, Cusabio, Wuhan, China, Cat. CSB-E04610r, and Muti Sciences, Cat. EK3825/2 for murine samples and Invitrogen, Cat. BMS6002, BMS618-3 and BMS607-2INST for cellular supernant samples respectively) according to the manufactural instructions.

### 4.9. Metabolomic Procedure and Data Processing

All of the plasma samples, 200 μL for each sample, were added methanol (600 μL, Methanol (HPLC), Fisher Chemical, Cat. A452-1) to make free of protein. Then the supernatant (500 μL) was lyophilized at −60 °C and 10.0 pa. The residue was re-dissolved in 100 μL of methanol-water (4:1, *v*/*v*). An aliquot of 2 μL was injected for UPLC-MS/MS analysis with Waters ACQUITY UPLC system (Waters Corporation, Milford, MA, USA), which had been equipped with a BEH C18 column (2.1 mm × 100 mm, 1.7 mm, Waters Corporation). A Waters Xevo G2-S Quadrupole Time-Of-Flight (QTOF) mass spectrometer (Waters Corporation) coupled to the UPLC system was utilized to carry out the mass spectrometry with an electrospray ionization in both and positive (ESI^+^) and negative (ESI^−^) ion modes. The chromatographic conditions and spectrometric parameters were set and optimized, as described previously [51].

To ensure the stability and suitability of MS analysis, a QC sample was prepared by pooling the same volume (20 μL) from every plasma samples. Ten chromatographic peaks of ions from the QC sequencing datasheet were selected to evaluate the validation of systematic method. Repeatability and intermediate precision on spectrum and chromatogram were also estimated with six successive QC injections or 6 replicates of a plasma in both ESI^+^ and ESI^−^ modes before the injection of the plasma samples. Moreover, four QC injections were performed randomly through the whole worklist.

The pre-processing of raw data was finished with MarkerLynx XS V4.1 software for alignment, deconvolution, and data reduction, so as to pair the mass and retention time with the corresponding intensities of all detected peaks. The main parameters were set similarly as before [51]. The processed files from ESI^+^ and ESI^−^ modes were exported for multivariate analysis.

### 4.10. Molecular Docking of Methotrexate within Caspase-1

Molecular docking of methotrexate within caspase-1 was performed with Schrödinger software packages. Maestro (version 2015-2 demo, Schrödinger) was used for protein and ligand preparation, receptor grid generation and docking. The X-ray crystal structure of inhibited interleukin-1β converting enzyme (Protein Data Bank (PDB) code: 1IBC) was retrieved and downloaded from the PDB database (available online: http://www.rcsb.org/pdb). Caspase-1 protein was prepared in the Protein Preparation Wizard to optimize the structure with assigning bond orders and water orientations, removing water molecules, adding hydrogens, creating zero-order bonds to metals, and disulfide bonds. Energy of the protein was minimized with the default parameters. Receptor grid of the protein ligand was generated with the limited size of 20 Å at the active site. The crystal structure of ligand, methotrexate, was downloaded from National Center for Biotechnology Information Open Chemistry Database (NCBI, PubChem CID: 126941), and optimized in the Ligand Preparation Wizard prior to the molecular docking. Docking was performed on the Workspace using GLIDE v.6.7. At least 10 conformations were set for methotrexate. The docking result was plotted with Pymol 1.8 software, which was based on python 2.7.

### 4.11. Statistic and Bioinformatic Analysis

Multivariate analysis for metabolomics data pre-possessed was finished with SIMCA-P software (v14.1, Umetric, Umeå, Sweden). PCA and OPLS-DA models were established. Variable importance of project (VIP) values, which indicated a significant difference between the groups were also estimated statistically. RSD also was calculated for the pooled QC injections to assess the quality and stability of MS data. All the distinct metabolites were identified by matching accurate mass (ppm < 10) to the Human Metabolome Database (HMDB Version 4.0) [52], with confirmation determined by comparing characteristic tandem mass spectrometry (MS/MS) fragmentation patterns with METLIN [53] and HMDB Database [52] or demonstrated by referring the chemical standards (HPLC grade, Sigma). All distinct metabolites were analyzed with MetaboAnalyst 4.0 for metabolic pathways [54]. The comprehensive metabolic network was constructed with Cytoscape Software (v3.6.1) [55] based on the data from Kyoto Encyclopedia of Genes and Genomes (KEGG) database [56].

Statistical significance of the difference was estimated using the F-test. Kolmogorov-Smirnov test was used to ensure the normality of the data. Student’s t test for data with homogeneity of variance or Welch’s *t*-test was performed for pairwise two-group analysis. Moreover, multiple comparisons among the groups were performed by one-way analyses of variance (ANOVA). Mann-Whitney-Wilcoxon test was performed for the dataset, which does not follow the normality. All statistical significance was accepted at *p* < 0.05. The statistical analysis was completed with R (v3.3.3) basic statistical packages. All of the statistical bar charts were finished with R’s Basic Graphic and ggplot2 (v2.2.1) packages. The heatmap of all the differentiated metabolites were prepared with ComplexHeatmap (v1.19.1) package. The specificity and sensitivity of biomarkers was displayed by ROC estimated with pROC (v1.12.1) package.

## 5. Conclusions

We demonstrated that methotrexate play an inhibitory effect on rheumatoid arthritis swelling and inflammation by downregulating NF-κB and NLRP3/Caspase-1 pathways and potentially affecting the activity of caspase-1. The critical perturbed metabolic pathways, including arachidonic acid, linoleic acid, and sphingolipid interacting with NF-κB and NLRP3/Caspase-1 could contribute to the pathogenesis of RA and were also involved in the pharmacological regulation of methotrexate. Various differentiated mediators provide us with series potential clinical biomarkers for future investigation. The elucidating pharmacological mechanism of methotrexate will benefit its rational administration in clinical practice.

## Figures and Tables

**Table 1:** Detailed results of all differentiated compounds identified in plasma.

NO	tR/min	Mass/Da	ESI ^§^	VIP	Identified Compound	KEGG ID	PPM	MS^E^ Fragmentation ^†^	Pathways
S1 *	17.26	295.2306	−	1.47	9,10- & 12,13-EpOME	C14825 ^▲^	9	134.8979, 152.9976, 167.1678, 183.0139, 195.8134, 197.2099, 223.1726, **277.2354**, **295.2302**	Linoleic acid
S2	18.15	303.2299	+	2.47	Linoleic acid	C01595	1	105.1102, 146.9803, **149.0211**, 165.0605, 185.0739, 221.9592, 281.1889	Linoleic acid
S3	22.98	303.2358	−	1.43	Arachidonic acid	C00219	9	100.9381, 160.8434, 219.8489, 243.9024, 255.2361, 259.2451, **303.2365**	Arachidonic acid
S4 *	18.26	319.2309	−	1.02	EETs ^#^ & HETEs ^##^	C14768 ^▲▲^	8	114.9385, 127.4831, 136.9957, 163.1423, 174.9591, 219.1486, 235.1270, 255.6255, 257.6209, 319.2300, etc.	Arachidonic acid
S5	29.05	341.1083	−	2.26	Alpha-lactose	C00243	2	116.9300, **145.1682**, **160.8460**, 265.1563, 277.4372, 281.2538, 311.1749	Starch and Galactose
S6	29.24	341.1122	−	2.01	Sucrose	C00089	2	101.0466, 146.9683, 160.9446, **249.5477**, 341.1111	Starch and Galactose
S7	29.23	387.1167	−	1.25	Trehalose	C01083	6	101.0466, 146.9683, 160.9446, **179.0878**, 341.1111	Starch and Galactose
S8 *	19.10	397.2239	−	1.15	Prostanoids	C02198 ^▲▲▲^	2	115.1325, **134.9978**, 166.8685, 183.0141, 205.5410, 207.1378, 279.2340, 303.2343, 351.1919	Arachidonic acid
S9	18.69	624.3016	−	1.12	Leukotriene C4	C02166	9	146.1692, 167.0458, **225.0720**, 508.3372, 562.3419	Arachidonic acid
S10 *	2.55	648.6284	−	1.13	Ceramide (d18:1/24:0)	C00195	2	**248.9640**, 316.9509, 452.9229, 649.8531	Sphingolipid
S11	24.76	730.5941	+	1.97	Sm(d18:0/18:1)	C00550	6	149.0213, **183.0713**, 282.2614, 310.2769, 561.3276	Sphingolipid
S12	25.75	850.5526	−	1.82	Lactosylceramide (d18:1/12:0)	C01290	1	**146.9697**, 179.0561, 198.2738, 480.3100, 626.3899	Sphingolipid
S13	28.68	734.5693	+	2.97	Pc (16:0/16:0)	C00157	0	184.0714, **496.2644**, 551.5518, 734.5715	Linoleic acid & Glycerophospholipid
S14	26.62	742.5342	-	3.87	Pc (15:0/18:2)	C00157	7	**141.0705**, 279.2360, 281.2520, 655.4543, 742.5328	
S15	27.63	752.5248	+	2.78	Pc (16:1/16:1)	C00157	6	184.0159, **237.0634**, 279.2367, 281.0246, 464.3080, 474.2838	
S16	24.79	756.5538	+	1.85	Pc (14:0/20:3)	C00157	0	183.0151, 247.2348, 271.2355, **468.3029**, 628.3584	
S17	23.84	758.5713	+	2.89	Pc (18:1/16:1)	C00157	2	184.0157, 255.2365, 590.5201, 655.4091, **740.5466**	
S18	26.38	782.5714	+	3.71	Pc (20:4/16:0)	C00157	3	184.0706, 257.2607, **478.3263**, 679.5030	
S19	27.41	786.6048	+	18.77	Pc (18:1/18:1)	C00157	5	184.0718, 283.2617, 339.3408, 604.5342, **786.6055**	
S20	27.08	798.5323	−	2.59	Pc (20:4/14:0)	C00157	4	227.2040, 257.2359, **303.2356**, 439.2255, 669.4767	
S21	26.90	804.5745	−	1.42	Pc (14:1/20:0)	C00157	2	181.1628, **265.2368**, 307.2393, 758.5609	
S22	28.72	810.6019	+	23.25	Pc (20:3/18:1)	C00157	1	184.0713, 221.0816, 283.2609, 307.2764, **627.3745**	
S23	25.79	832.5876	+	6.78	Pc (18:2/20:2)	C00157	6	184.0728, 309.2785, 627.3777, 684.4404, **752.5748**	
S24	26.87	854.5839	−	4.99	Pc (20:4/18:0)	C00157	9	255.2352, 279.2362, 283.2639, 303.2360, **725.5103**	

^§^ ESI modes, Positive mode (+) and Negative mode (−) of electrospray ionization; ^†^ Spectrum ion mass fragments in MS/MS under MS^E^ mode. The characteristic fragments are highlighted as bold font; * Metabolites identified against to the standard chemicals; ^#^ EET, epoxyoctadecenoic acid; ^##^ HETE, hydroxyeicosatetraenoic acid; ^▲^, include not only C14825, but also C14826; ^▲▲^, include not only C14768, but also C14769, C14771, C04742 and C04805; ^▲▲▲^, include not only C02198, but also C00696.

**Table 2:** Molecular docking values of methotrexate within caspase-1.

Items	Potential Energy-OPLS3	Docking Score	XP GScore	H-Bond
Values	209.403	−4.683	−5.953	6

## Supplementary Materials

Supplementary materials can be found at http://www.mdpi.com/1422-0067/19/10/2894/s1.

## Abbreviations

ANOVA	Analysis of Variance
ARG	Arginine
ATCC	American Type Culture Collection
AUC	Area Under Curve
CIA	Collagen induced arthritic
DCS	Diclofenac sodium
DMEM	Dulbecco’s Modified Eagle’s Medium
ECL	Chemiluminescence
EET	Epoxyoctadecenoic acid
EpOME	Epoxide of linoleic acid
ESI	Electrospray Ionization
FBS	Fetal Bovine Serum
HETE	Hydroxyeicosatetraenoic acid
HMDB	Human Metabolome Database
HRP	Horseradish Peroxidase
IOD	Integrated optical density
KEGG	Kyoto Encyclopedia of Genes and Genomes
LPS	Lipopolysaccharide
LTC4	Leukotrienes C4
MS	Mass Spectrometry
MS/MS	tandem mass spectrometry
NF-κB	Nuclear factor kappa-light-chain-enhancer of activated B cells
NLRP3	Nucleotide binding domain and leucine-rich repeat pyrin 3 domain
NSAIDs	Nonsteroidal anti-inflammatory drugs
OPLS-DA	Orthogonal Projections to Latent Structures Discriminant Analysis
PC	Principle Component
PCA	Principle Component Analysis
Pc	Phosphatidylcholine
PDB	Protein Data Bank
PDG2	Prostaglandin D2
PPM	Mass error ppm
PVDF	Polyvinylidene Difluoride
Q/TOF	Quadrupole time of flight mass spectrometry
QC	Quality Control
RA	Rheumatoid arthritis
ROC	Receiver Operating Characteristic Curve
RSD	Relative Standard Deviation
RTCA	Real Time Cellular Analysis
sPBS	Stimulated with LPS followed by PBS
SM	Sm(d18:0/18:1)
TXA2	Thromboxane A2
UPLC	Ultra-Performance Liquid Chromatography
VIP	Variable Importance for the Projection
