# Supplementary material for: Inhibitory Effect of Methotrexate on Rheumatoid Arthritis Inflammation and Comprehensive Metabolomics Analysis Using Ultra-Performance Liquid Chromatography-Quadrupole Time of Flight-Mass Spectrometry (UPLC-Q/TOF-MS)
Source: Int J Mol Sci. 2018 Sep 23;19(10):2894. doi: 10.3390/ijms19102894 (PMC6212996; doi:10.3390/ijms19102894)
Supplement: Supplementary file 1 [file ijms-19-02894-s001.pdf]

SUPPLEMENTARY MATERIALS

Inhibitory Effect of Methotrexate on Rheumatoid Arthritis Inflammation and Comprehensive Metabolomics Analysis Using UPLC-Q/TOF-MS

Zhiqiang Pang [1], Guoqiang Wang [1], Nan Ran [1], Hongqiang Lin [2], Ziyan Wang [1], Xuewa Guan [1], Yuze Yuan [1], Keyong Fang [3], Jinping Liu [2] and Fang Wang [1,*]

[1] Department of Pathogen Biology, College of Basic Medical Sciences, Jilin University, Changchun, China; pangzq2812@mails.jlu.edu.cn (Z.P.); wanggq14@mails.jlu.edu.cn (G.W.); rannan17@mails.jlu.edu.cn (N.R.); wzy16@mails.jlu.edu.cn (Z.W.); guanxw15@mails.jlu.edu.cn (X.G.); yuanyz17@mails.jlu.edu.cn (Y.Y.)

[2] Research Center of Natural Drug, School of Pharmaceutical Sciences, Jilin University, Changchun, China; linhq17@mails.jlu.edu.cn (H.L.); liujp@jlu.edu.cn (J.L.)

[3] Department of Pharmacology, College of Basic Medical Sciences, Jilin University, Changchun, China; fangky15@mails.jlu.edu.cn (K.F.)

* Correspondence: wf@jlu.edu.cn; Tel.: +86-431-85168587

Table S1 Investigation on the stability and suitability of chromatographic and spectrometric system

Modes	NO	Retention Time/min	m/z	m/z in SPECTRUM		RT of CHROMATOGRAM		PA of CHROMATOGRAM	
				Repeatability (RSD%)	Precision (RSD%)	Repeatability (RSD%)	Precision (RSD%)	Repeatability (RSD%)	Precision (RSD%)
ESI+	1	0.5788	203.0535	0.0006	0.0003	0.0012	0.0000	2.7081	5.2620
	2	12.7089	274.2727	0.0000	0.0001	0.0001	0.0000	1.1709	4.6712
	3	9.7526	347.2203	0.0001	0.0002	0.0215	0.1777	3.1600	2.9135
	4	27.1474	416.2668	0.0001	0.0001	0.1037	0.0554	2.2565	3.1535
	5	20.6052	524.3746	0.0002	0.0002	0.0052	0.0000	3.4421	5.1513
	6	27.6191	663.4358	0.0001	0.0001	0.2473	0.6784	2.1183	5.7450
	7	27.6164	708.5138	0.0000	0.0002	0.1245	0.6980	1.7551	3.8983
	8	27.1398	803.5465	0.0000	0.0001	0.0120	0.0640	2.7071	5.7567
	9	17.9762	991.6768	0.0004	0.0008	0.1020	0.0835	3.9924	2.3065
	10	16.9942	1063.6670	0.0001	0.0004	0.0047	0.0882	1.6940	5.6129
ESI-	1	0.6089	215.0353	0.0000	0.0002	0.0034	0.0000	1.3124	1.0261
	2	24.8625	355.1600	0.0003	0.0002	0.0149	0.1612	2.2078	5.6903
	3	18.1080	480.3099	0.0001	0.0000	0.1674	0.2611	1.6232	4.9489
	4	17.5954	504.3313	0.0002	0.0001	0.0182	0.2048	3.4140	3.3804
	5	17.0883	588.3303	0.0001	0.0002	0.2045	0.2045	5.6507	3.6115
	6	20.7359	640.2898	0.0002	0.0002	0.1934	0.1934	0.1115	2.6260
	7	20.7430	711.2649	0.0000	0.0004	0.3050	0.3454	1.6040	5.0106
	8	10.3365	850.9446	0.0002	0.0002	0.0169	0.3832	1.1308	5.3971
	9	0.5257	928.8245	0.0000	0.0002	0.0000	0.0000	0.1076	4.1797
	10	18.1024	1035.6513	0.0000	0.0001	0.0132	0.2611	1.0888	5.0841

Table S2 Primers designed for qPCR in animal and cell experiments

Species	Genes	Forward Primers	Reverse Primers
Rat	NLRP3	CAGACCTCCAAGACCACGACTG	CATCCGCAGCCAATGAACAGAG
	Caspase-1	TGCCTGGTCTTGTGACTTGGAG	ATGTCCTGGGAAGAGGTAGAAACG
	NF-κB p65	CTCACCGGCCTCATCCACAT	TGGCTAATGGCTTGCTCCAG
	TNF-α	GGCATGGATCTCAAAGACAACC	AAATCGGCTGACGGTGTGG
	IL-1β	TCTCACAGCAGCATCTCGAC	GGTCGTCATCATCCCACGAG
	IL-18	CGCAGTAATACGGAGCATAAATGAC	GGTAGACATCCTTCCATCCTTCAC
	β-actin	ACCACCATGTACCCAGGCATT	CCACACAGAGTACTTGCGCTCA
Mice (RAW 264.7)	NLRP3	ATTACCCGCCCGAGAAAGG	TCGCAGCAAAGATCCACACAG
	Caspase-1	TGCCTGGTCTTGTGACTTGGAG	ATGTCCTGGGAAGAGGTAGAAACG
	NF-κB p65	AGCTCAAGATCTGCCGAGTAA	GCCTGGTCCCGTGAAATACA
	TNF-α	GGCATGGATCTCAAAGACAACC	AAATCGGCTGACGGTGTGG
	IL-1β	TGAAAACACAGAAGTAACGTCCG	CCCAGGAGGAAATTGTAATGGGA
	IL-18	AATCACTTCCTCTTGGCCCA	GTTGTACAGTGAAGTCGGCC
	β-actin	AAGACCTCTATGCCAACACAGT	AGCCAGAGCAGTAATCTCCTTC

Table S3 MetaboAnalyst Results on Metabolic Pathways

	Total	Expected	Hits	Raw *P*	=-LOG(*P*)	Holm adjust	FDR	Impact
Arachidonic acid metabolism§	36	0.48787	10	2.57E-12	26.687	2.08E-10	2.08E-10	0.40905
Linoleic acid metabolism§	5	0.06776	4	1.20E-07	15.937	9.59E-06	4.85E-06	1
Sphingolipid metabolism§	21	0.28459	3	0.0024081	6.0289	0.19024	0.06502	0.2807
Starch and sucrose metabolism	23	0.3117	2	0.037171	3.2922	1	0.75272	0
Galactose metabolism	26	0.35235	2	0.046613	3.0659	1	0.75513	0.05678
alpha-Linolenic acid metabolism	42	0.56919	2	0.10867	2.2194	1	1	0
Biosynthesis of unsaturated fatty acids	9	0.12197	1	0.11588	2.1552	1	1	0
Glycerophospholipid metabolism	30	0.40656	1	0.33878	1.0824	1	1	0.13889

§ Metabolic pathways significantly perturbed.

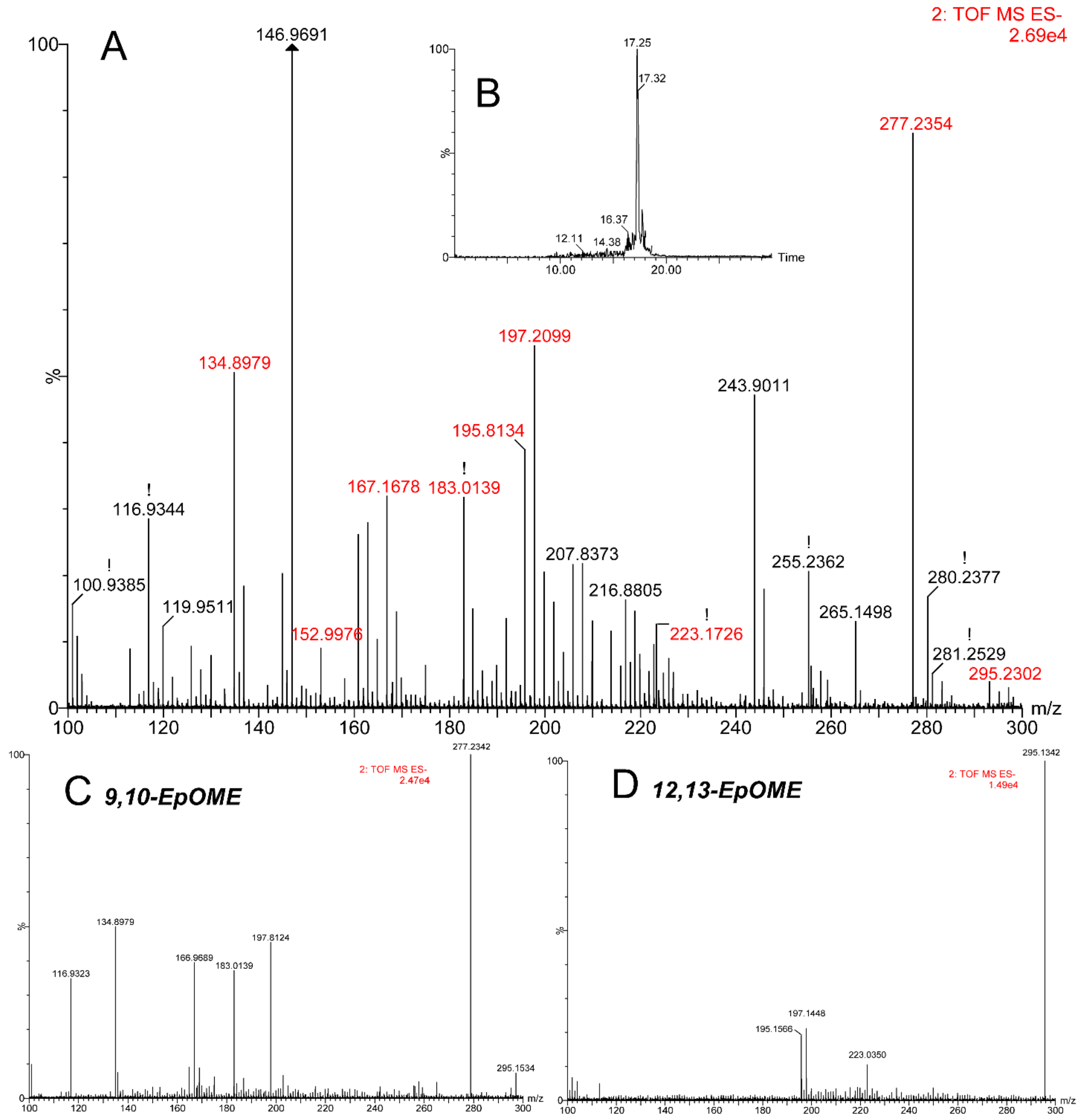

Figure S1. MS/MS spectra of the identified potential metabolic marker **S1** 9,10- & 12,13-EpOME (A) and the chromatographic peak of 9,10- & 12,13-EpOME (B). The reference standard chemical spectra including 9,10-EpOME (C), 12,13-EpOME (D) in experimental condition. The reference spectra from HMDB database with different voltage 20V (E) and 40V (F) for 9,10-EpOME and 20V (G) and 40V (H) for 12,13-EpOME.

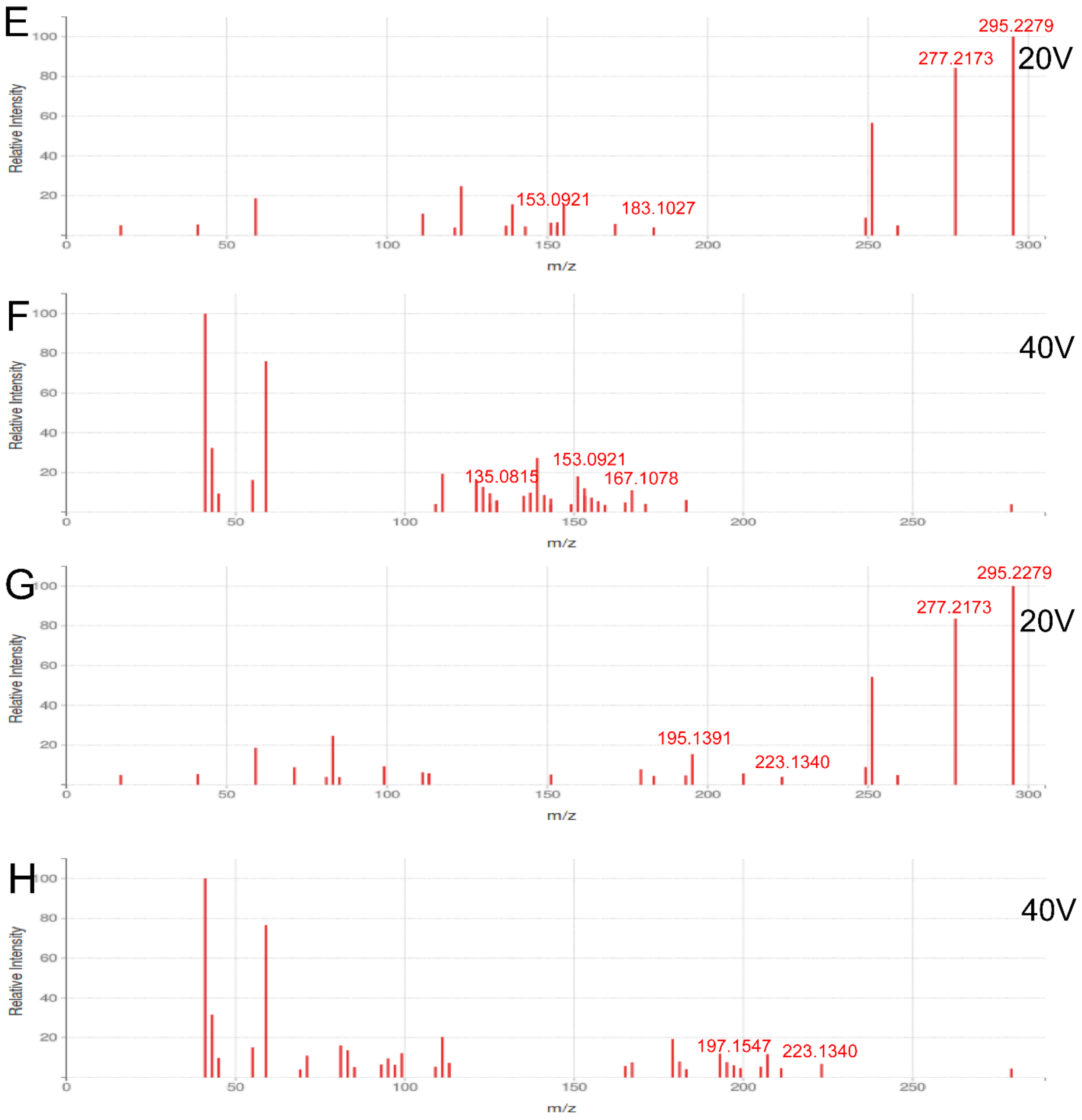

Figure S1. MS/MS spectra of the identified potential metabolic marker **S1** 9,10- & 12,13-EpOME (A) and the chromatographic peak of 9,10- & 12,13-EpOME (B). The reference standard chemical spectra including 9,10-EpOME (C), 12,13-EpOME (D) in experimental condition. The reference spectra from HMDB database with different voltage 20V (E) and 40V (F) for 9,10-EpOME and 20V (G) and 40V (H) for 12,13-EpOME.

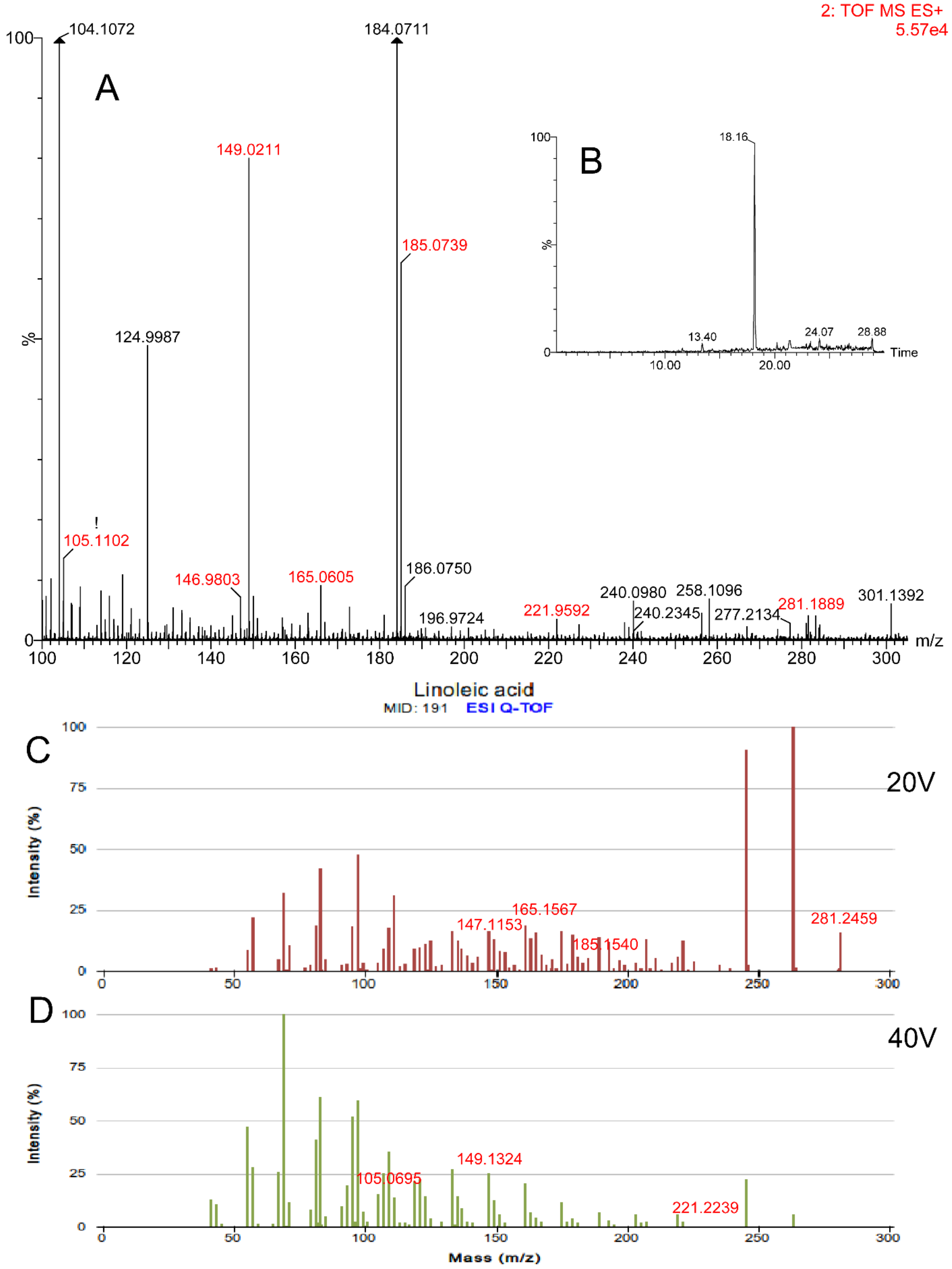

Figure S2. MS/MS spectra of the identified potential metabolic marker **S2** Linoleic acid (A) and the chromatographic peak of Linoleic acid (B). The reference spectra from METLIN database with different voltage 20V (E) and 40V (F).

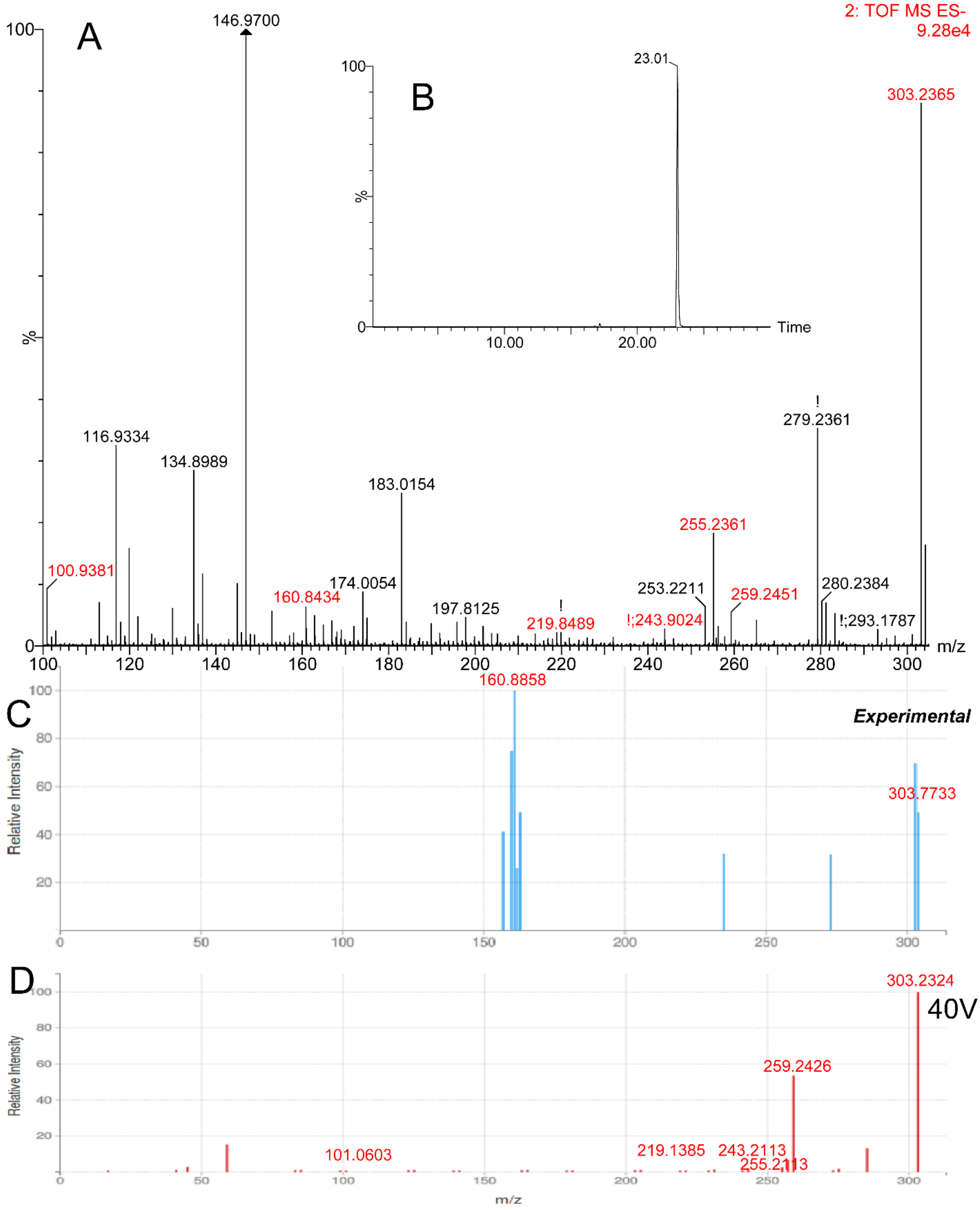

Figure S3. MS/MS spectra of the identified potential metabolic marker **S3** Arachidonic acid (A) and the chromatographic peak of Arachidonic acid (B). The reference spectra from HMDB database with in experimental condition (C) and voltage of 40V (D).

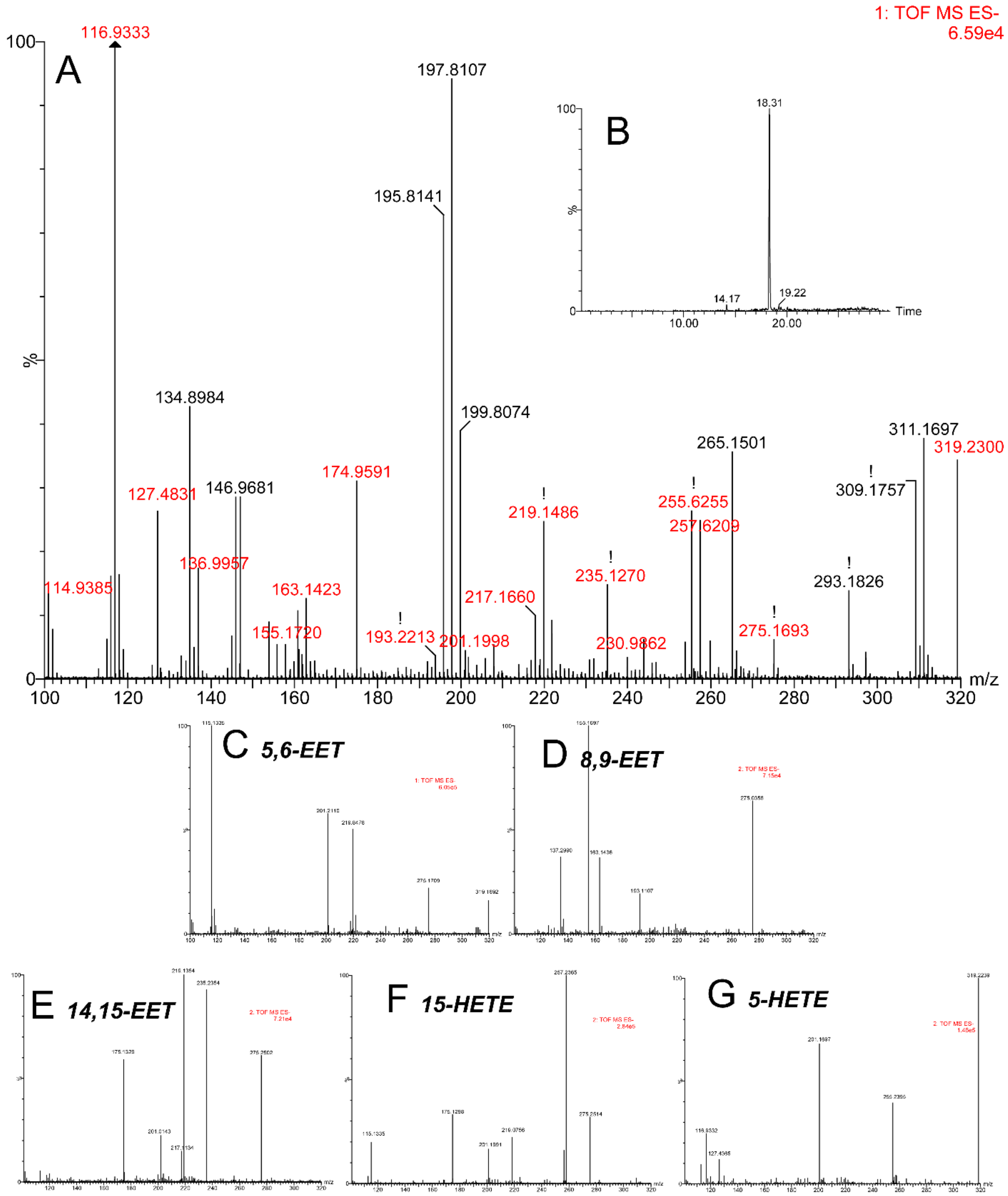

Figure S4. MS/MS spectra of the identified potential metabolic marker **S4** EETs & HETEs (A) and the chromatographic peak of EETs & HETEs (B). The reference standard chemical spectra including 5,6-EET (C), 8,9-EET (D), 14,15-EET (E), 15-HETE (F) and 5-HETE (G) in experimental condition. The reference spectra from HMDB database with voltage 20V for 5,6-EET (H), 8,9-EET (I), 14,15-EET (J), 15-HETE (K) and 5-HETE (L).

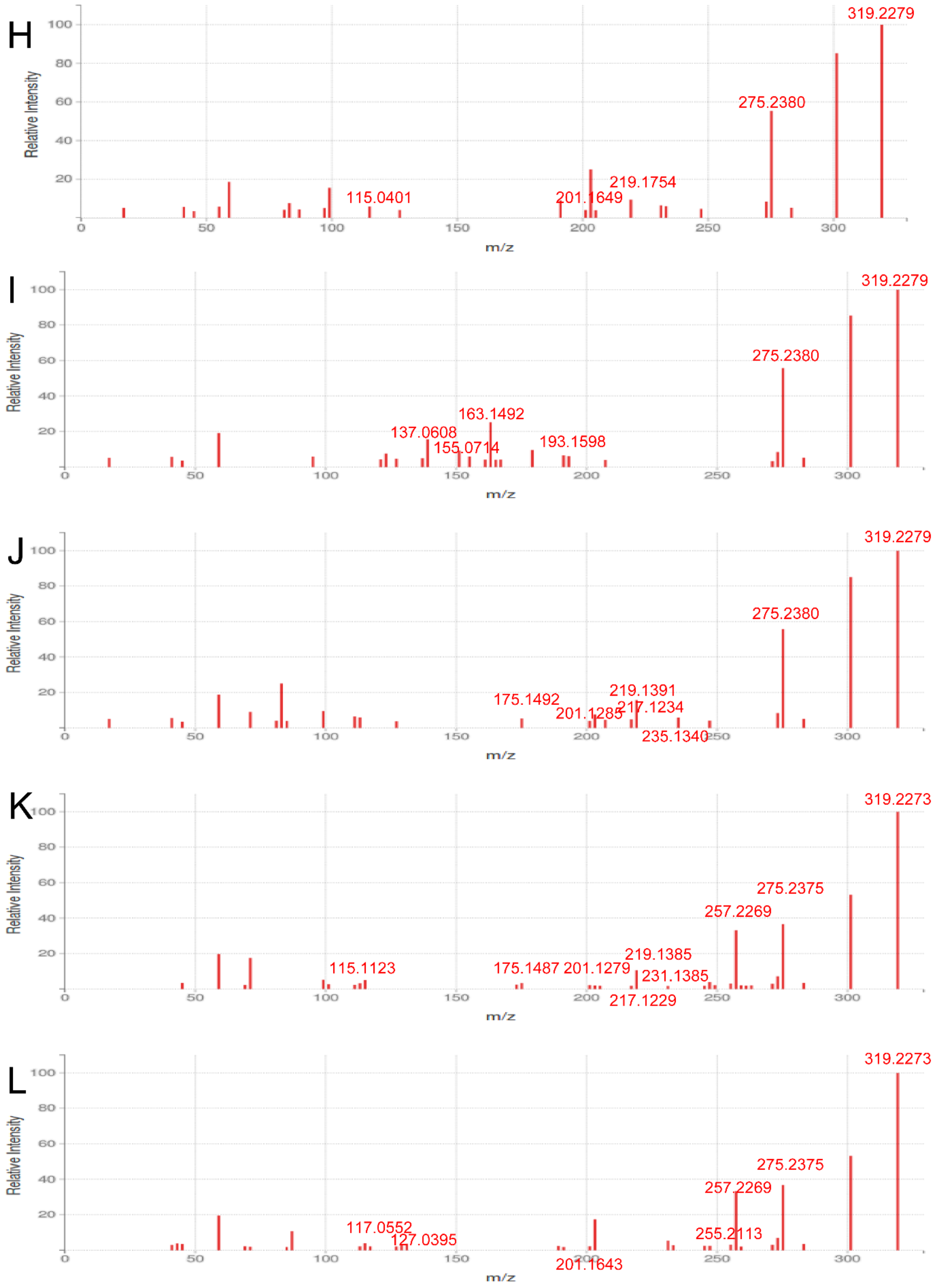

Figure S4. MS/MS spectra of the identified potential metabolic marker **S4** EETs & HETEs (A) and the chromatographic peak of EETs & HETEs (B). The reference standard chemical spectra including 5,6-EET (C), 8,9-EET (D), 14,15-EET (E), 15-HETE (F) and 5-HETE (G) in experimental condition. The reference spectra from HMDB database with voltage 20V for 5,6-EET (H), 8,9-EET (I), 14,15-EET (J), 15-HETE (K) and 5-HETE (L).

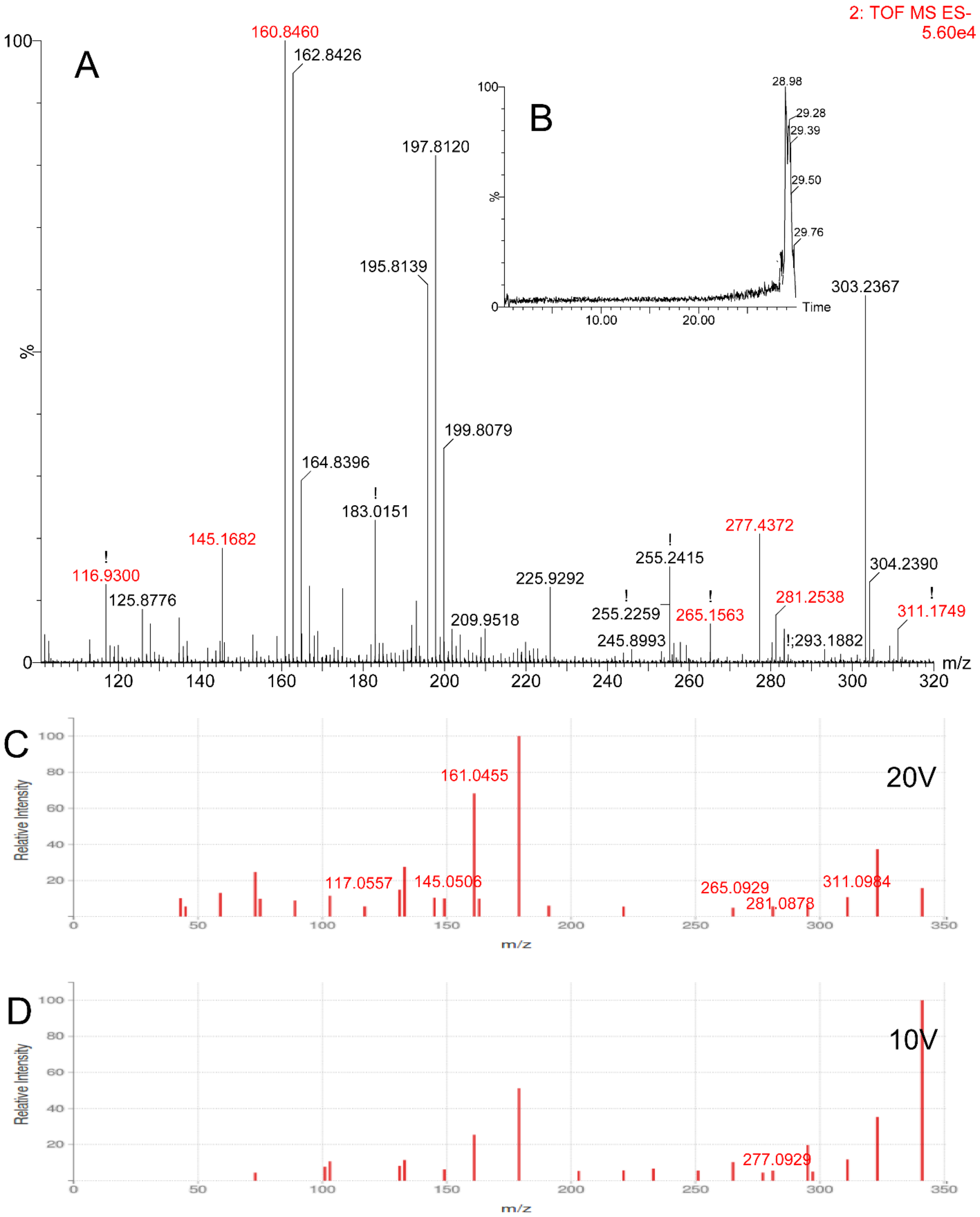

Figure S5. MS/MS spectra of the identified potential metabolic marker **S5** Alpha-lactose (A) and the chromatographic peak of Alpha-lactose (B). The reference spectra from HMDB database with in voltage 20V (C) and voltage of 40V (D).

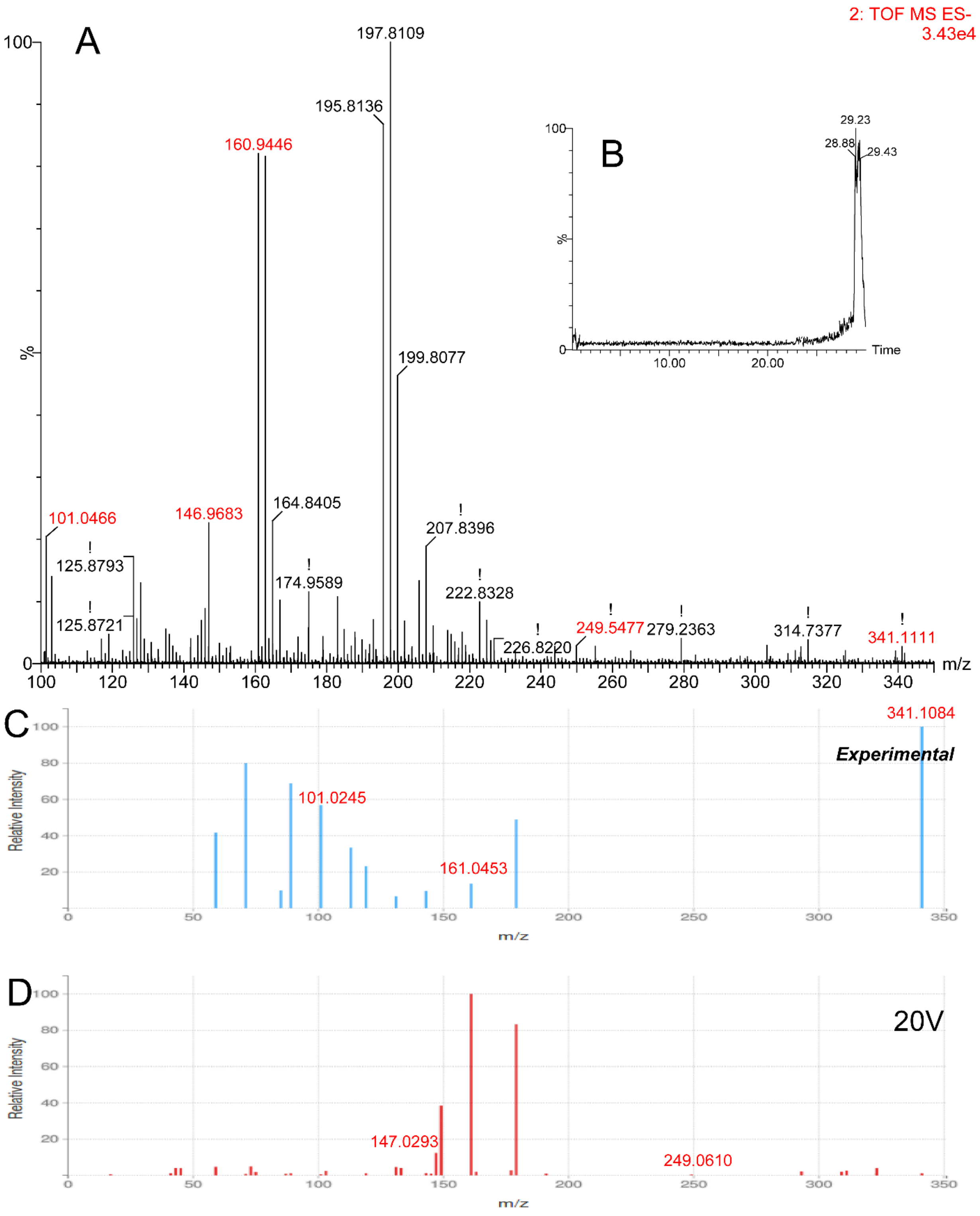

Figure S6. MS/MS spectra of the identified potential metabolic marker **S6** Sucrose (A) and the chromatographic peak of Sucrose (B). The reference spectra from HMDB database with in voltage 20V (C) and voltage of 40V (D).

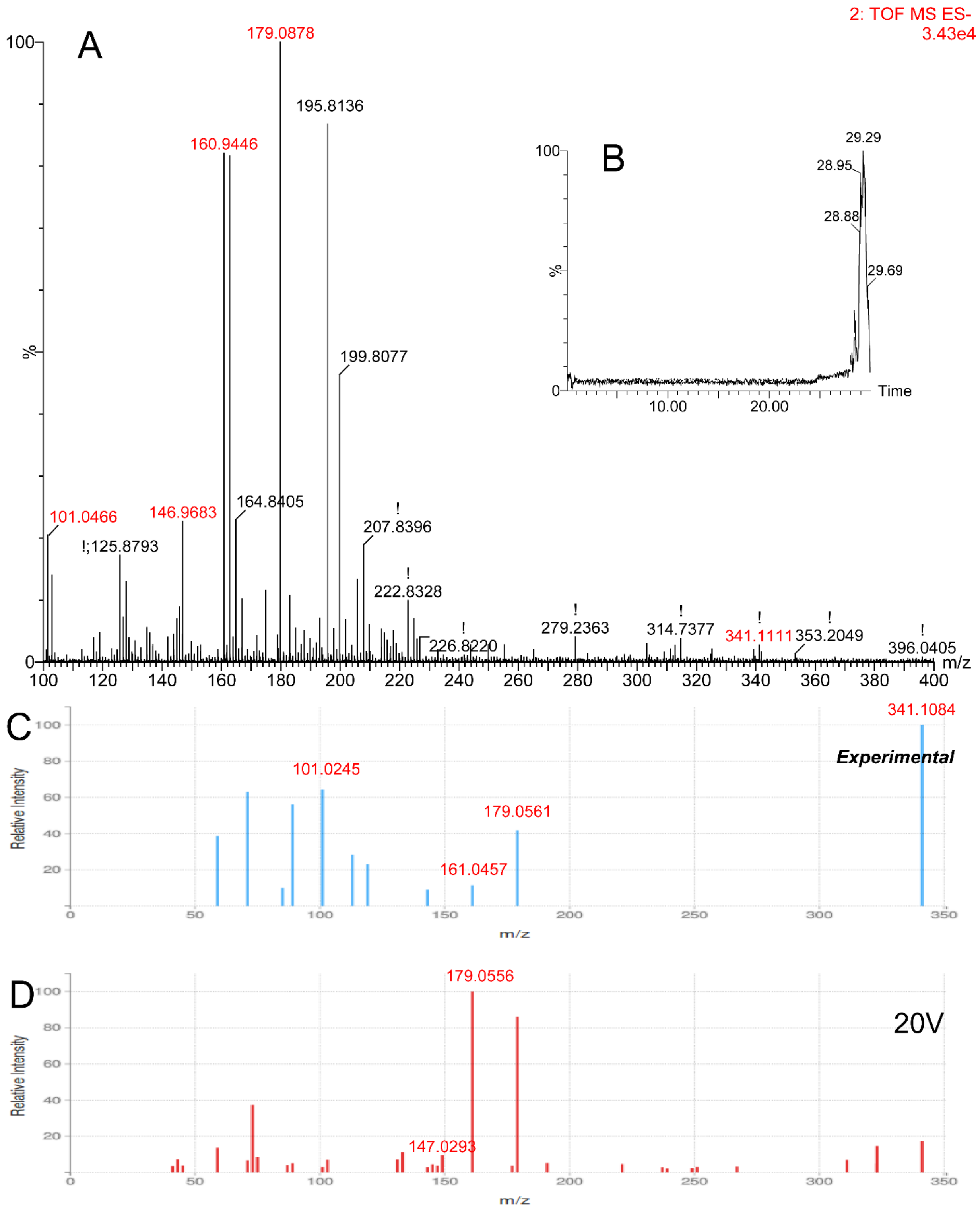

Figure S7. MS/MS spectra of the identified potential metabolic marker **S7** Trehalose (A) and the chromatographic peak of Trehalose (B). The reference spectra from HMDB database with in voltage 20V (C) and voltage of 40V (D).

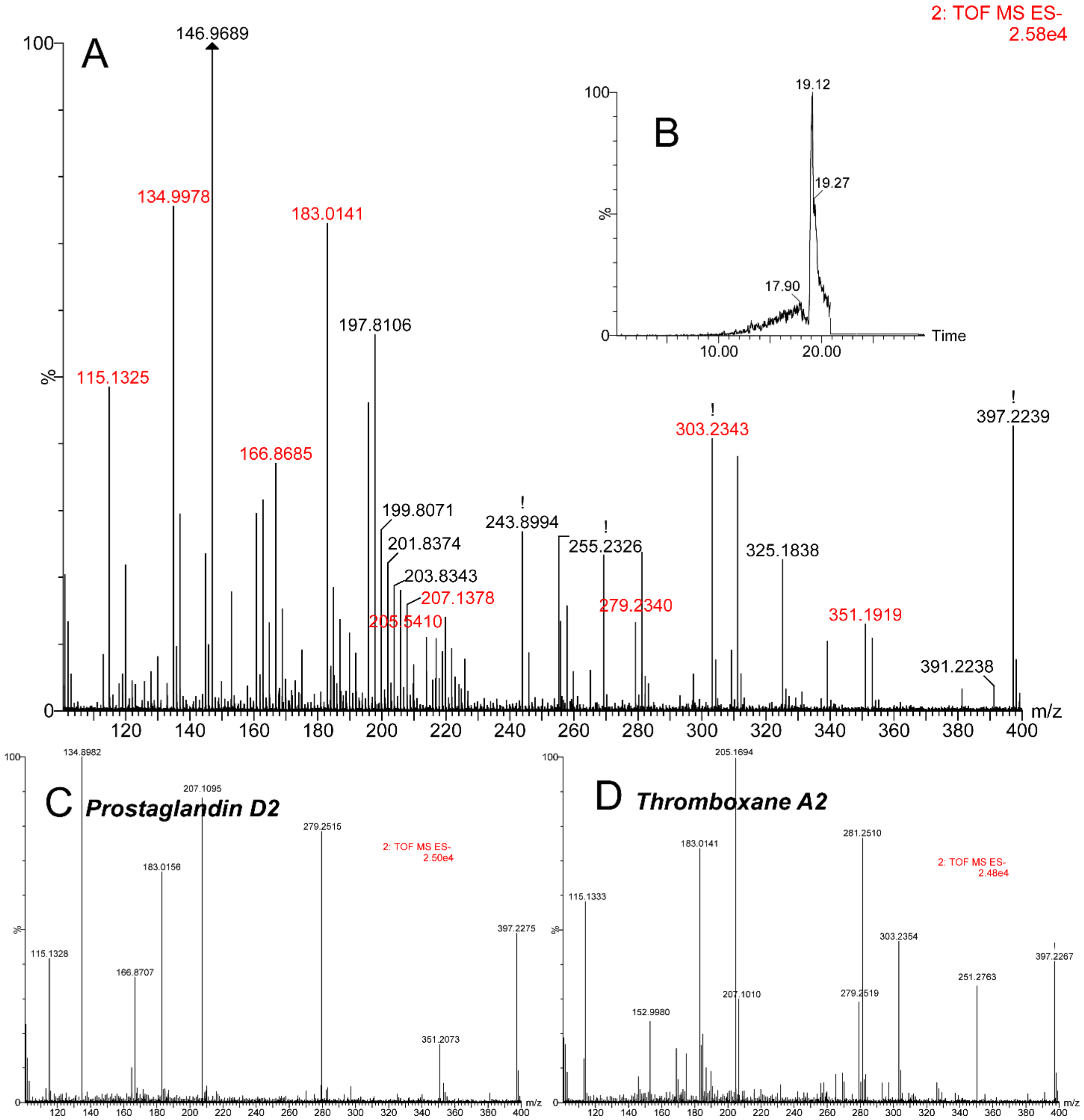

Figure S8. MS/MS spectra of the identified potential metabolic marker **S8** Prostanoids (A) and the chromatographic peak of Prostanoids (B). The reference standard chemical spectra including Prostaglandin D2 (C) and Thromboxane (D) in experimental condition. The reference spectra from HMDB database with voltage 20V for Prostaglandin D2 (E), 40V for Prostaglandin D2 (F) and 20V for Thromboxane (G).

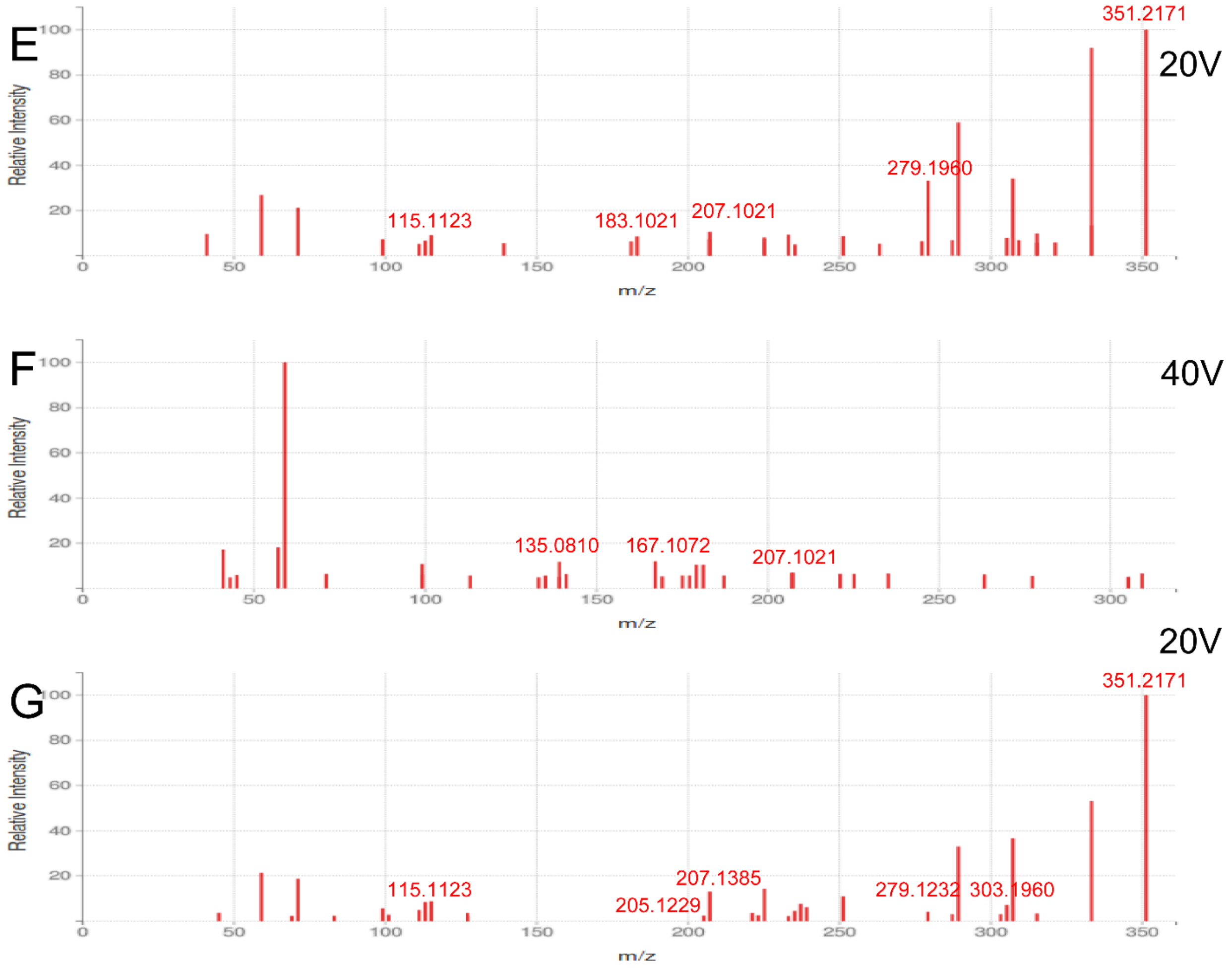

Figure S8. MS/MS spectra of the identified potential metabolic marker **S8** Prostanoids (A) and the chromatographic peak of Prostanoids (B). The reference standard chemical spectra including Prostaglandin D2 (C) and Thromboxane (D) in experimental condition. The reference spectra from HMDB database with voltage 20V for Prostaglandin D2 (E), 40V for Prostaglandin D2 (F) and 20V for Thromboxane (G).

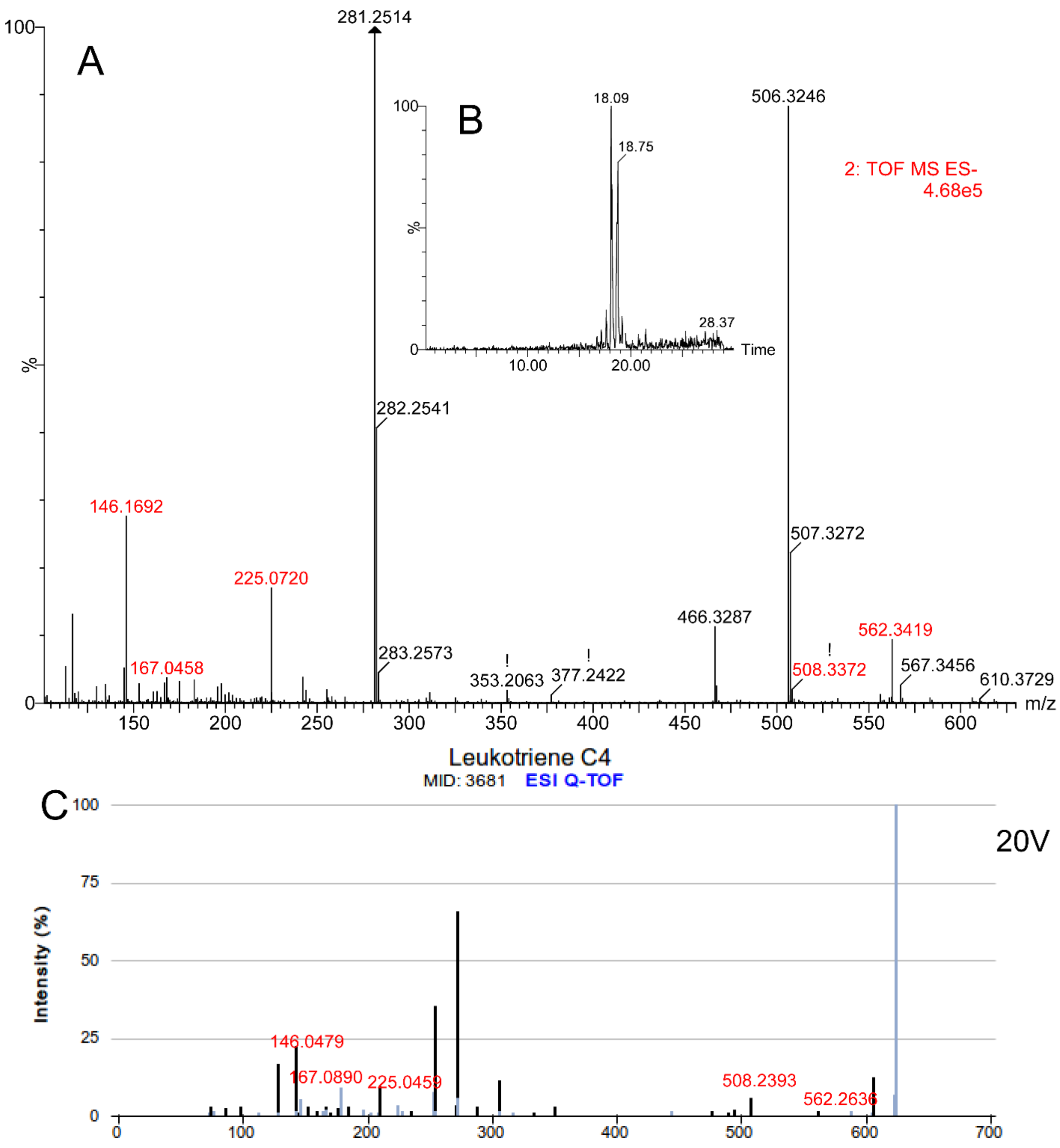

Figure S9. MS/MS spectra of the identified potential metabolic marker **S9** Leukotriene C4 (A) and the chromatographic peak of Leukotriene C4 (B). The reference spectra from METLIN database with in voltage 20V (C).

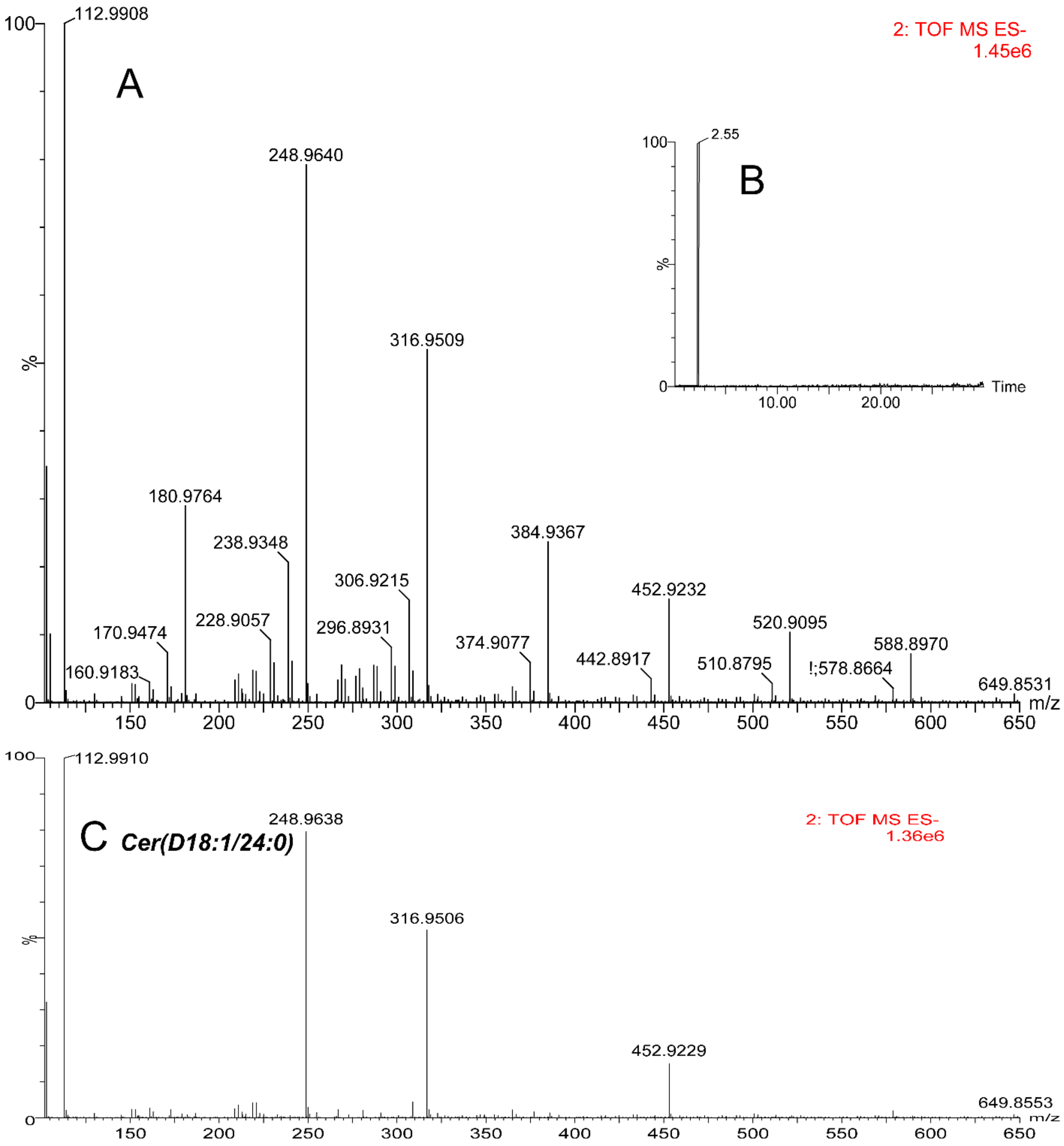

Figure S10. MS/MS spectra of the identified potential metabolic marker **S10** Ceramide (d18:1/24:0) (A) and the chromatographic peak of Ceramide (d18:1/24:0) (B). The reference standard chemical spectra of Ceramide (d18:1/24:0) (C).

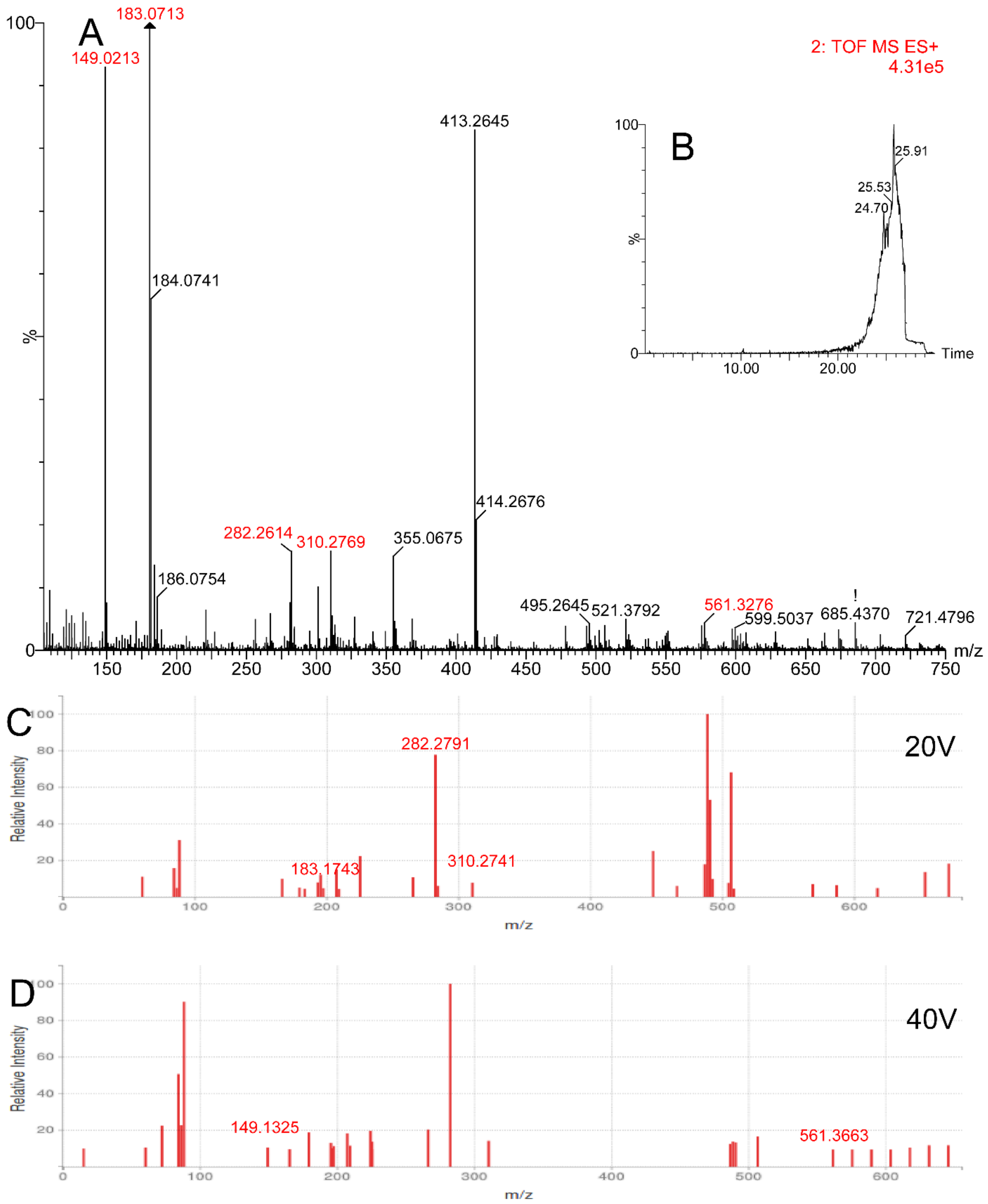

Figure S11. MS/MS spectra of the identified potential metabolic marker **S11** Sm(d18:0/18:1) (A) and the chromatographic peak of Sm(d18:0/18:1) (B). The reference spectra from HMDB database with in voltage 20V (C) and voltage of 40V (D).

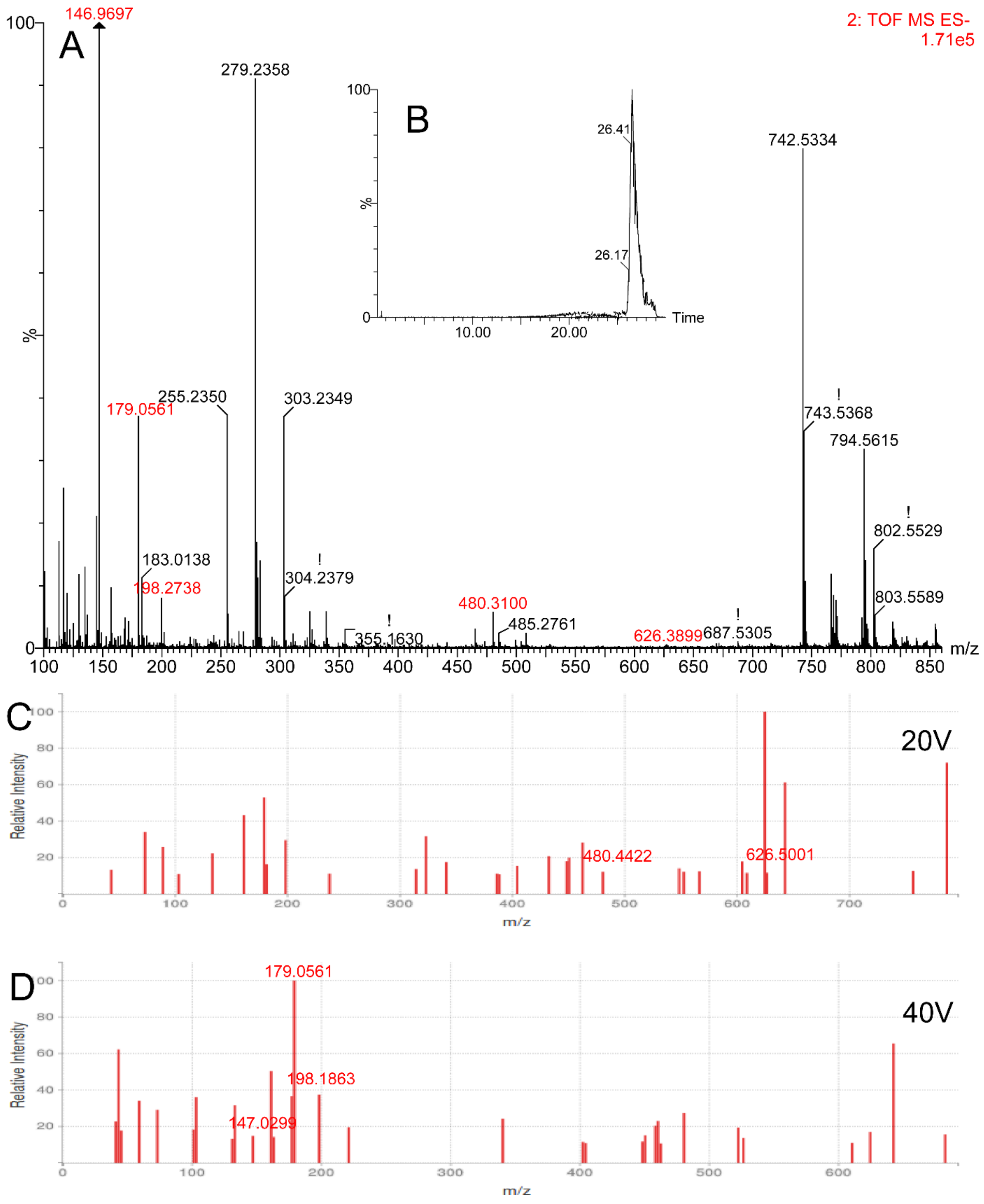

Figure S12. MS/MS spectra of the identified potential metabolic marker **S12** Lactosylceramide (d18:1/12:0) (A) and the chromatographic peak of Lactosylceramide (d18:1/12:0) (B). The reference spectra from HMDB database with in voltage 20V (C) and voltage of 40V (D).

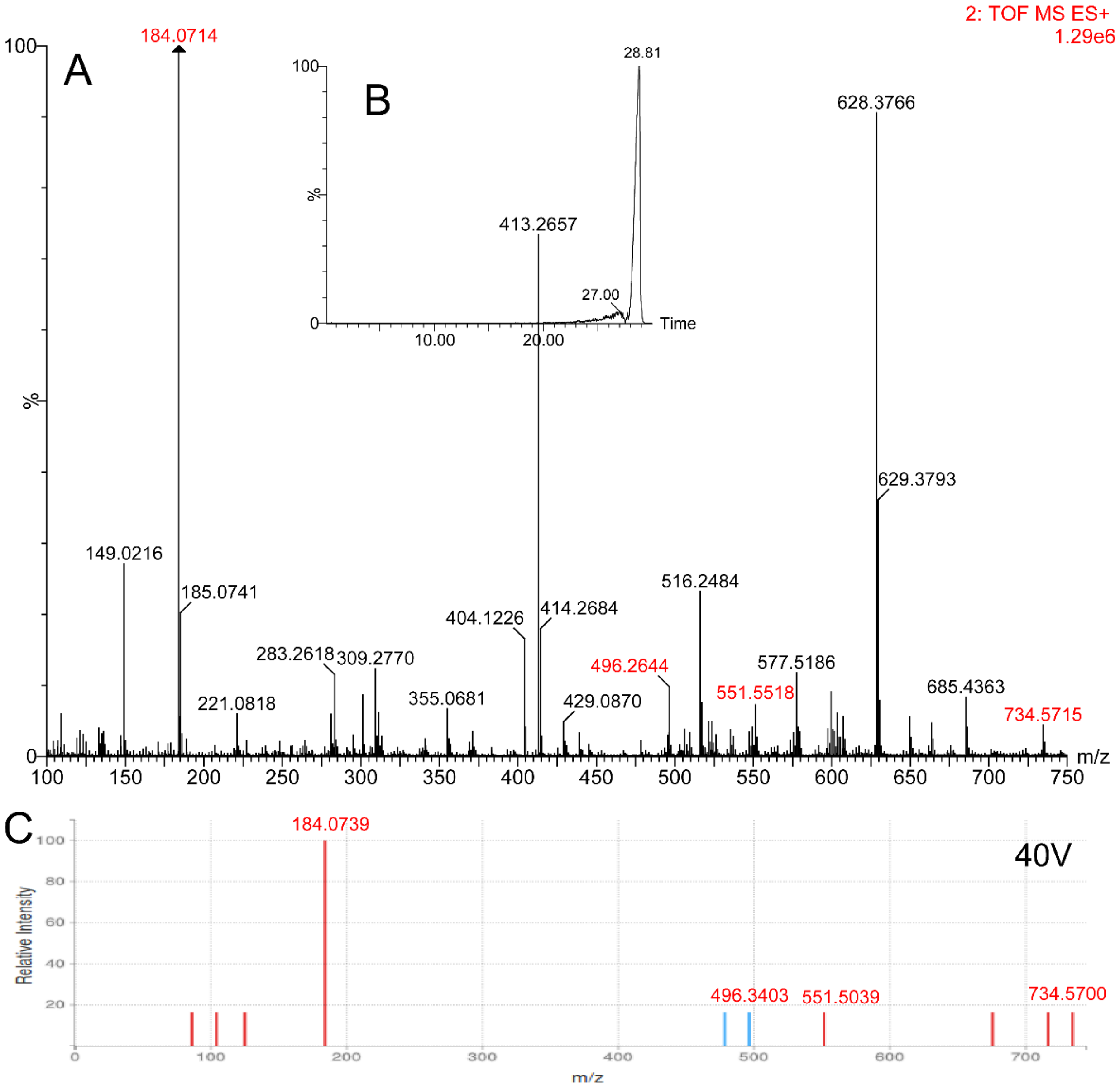

Figure S13. MS/MS spectra of the identified potential metabolic marker **S13** Pc(16:0/16:0) (A) and the chromatographic peak of Pc(16:0/16:0) (B). The reference spectra from HMDB database with in voltage 40V (C).

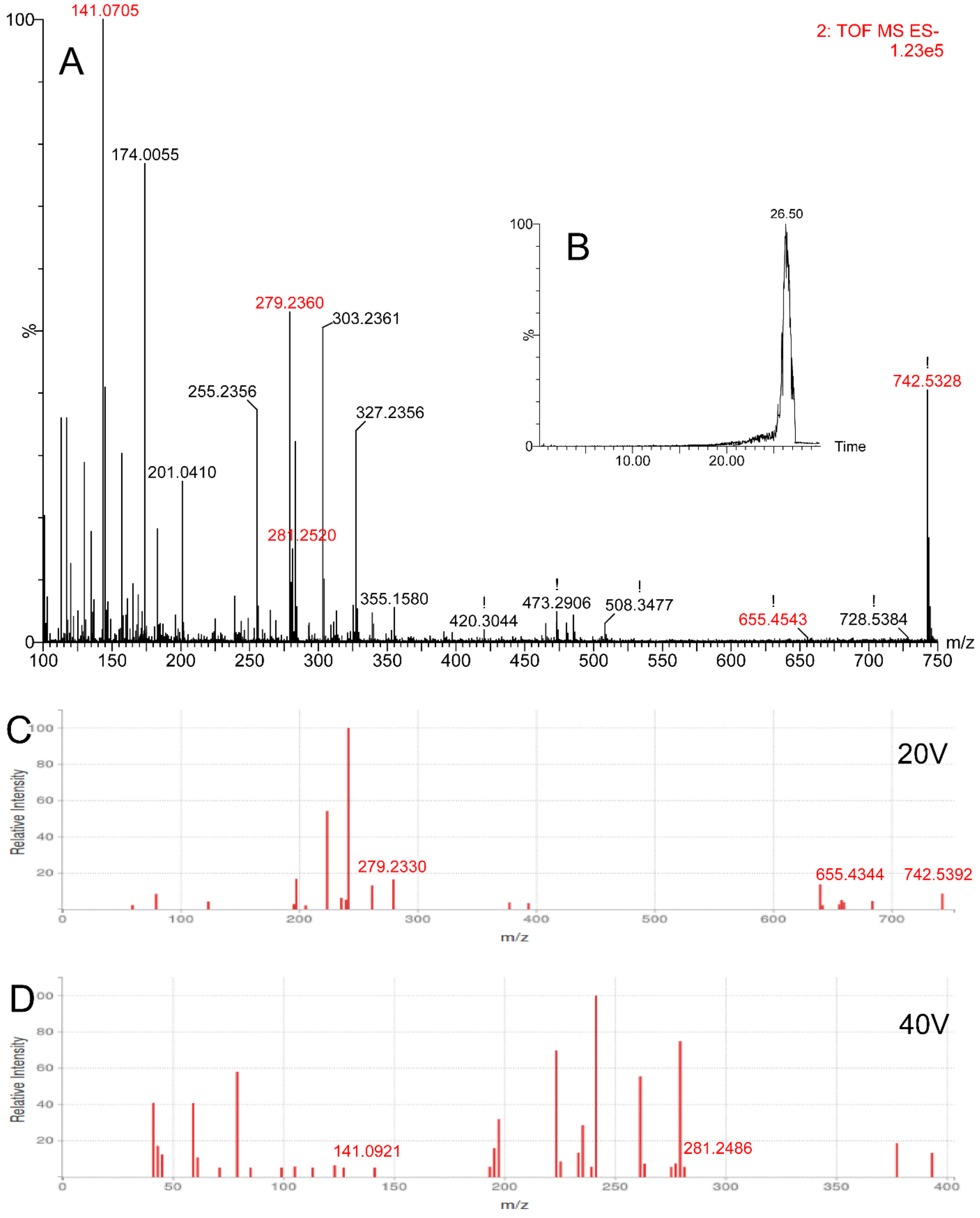

Figure S14. MS/MS spectra of the identified potential metabolic marker **S14** Pc(15:0/18:2) (A) and the chromatographic peak of Pc(15:0/18:2) (B). The reference spectra from HMDB database with in voltage 20V (C) and voltage of 40V (D).

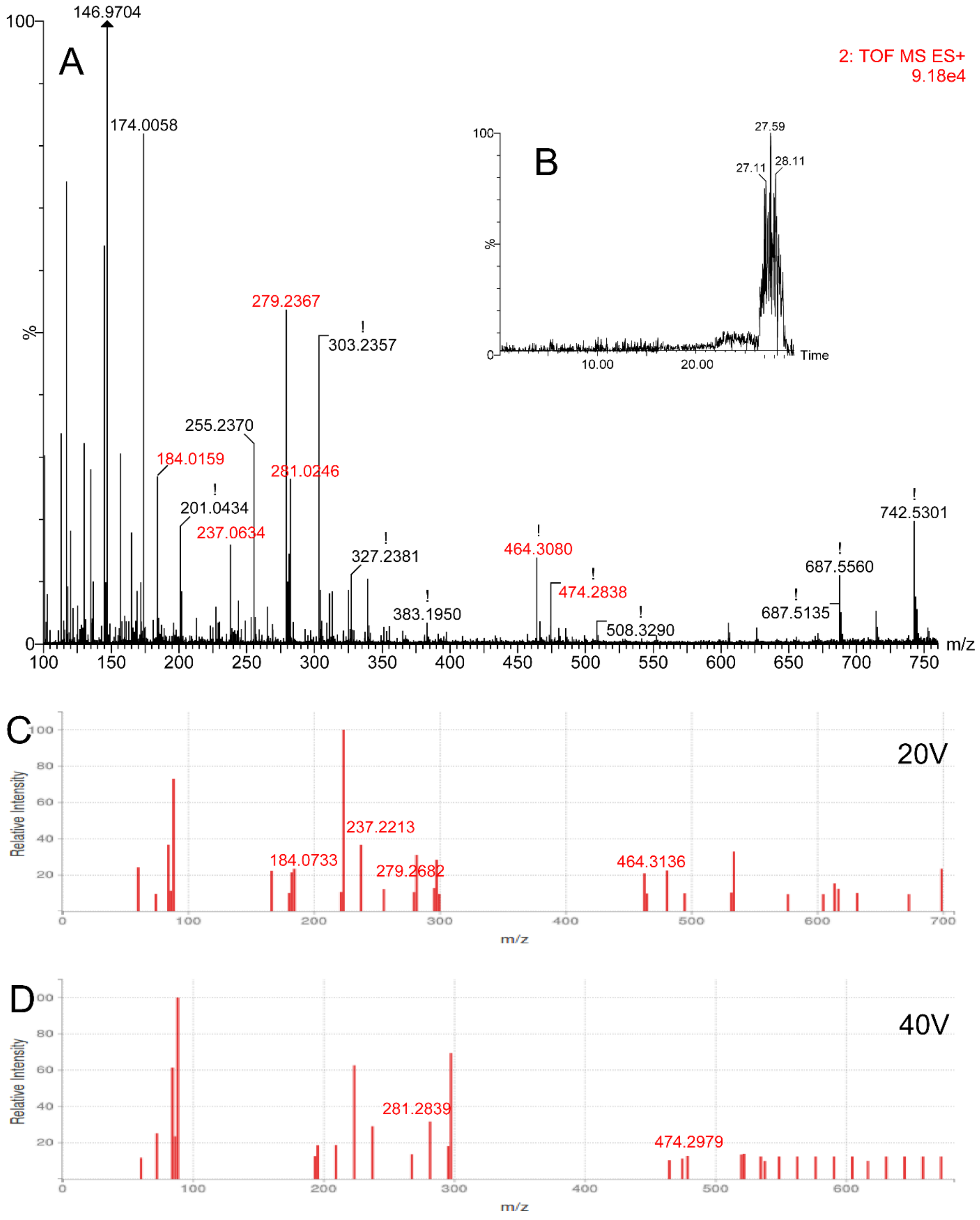

Figure S15. MS/MS spectra of the identified potential metabolic marker **S15** Pc(16:1/16:1) (A) and the chromatographic peak of Pc(16:1/16:1) (B). The reference spectra from HMDB database with in voltage 20V (C) and voltage of 40V (D).

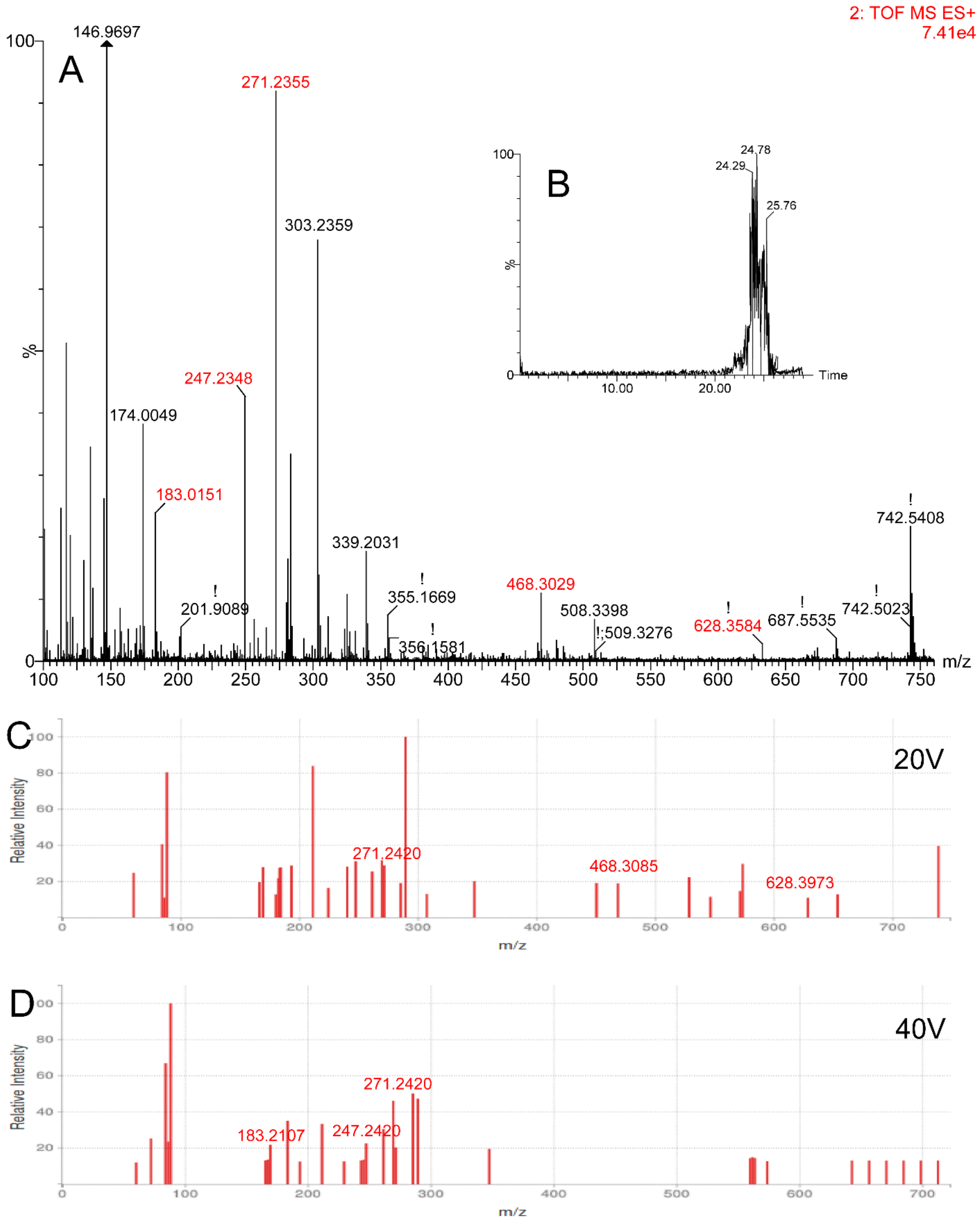

Figure S16. MS/MS spectra of the identified potential metabolic marker **S16** Pc(14:0/20:3) (A) and the chromatographic peak of Pc(14:0/20:3) (B). The reference spectra from HMDB database with in voltage 20V (C) and voltage of 40V (D).

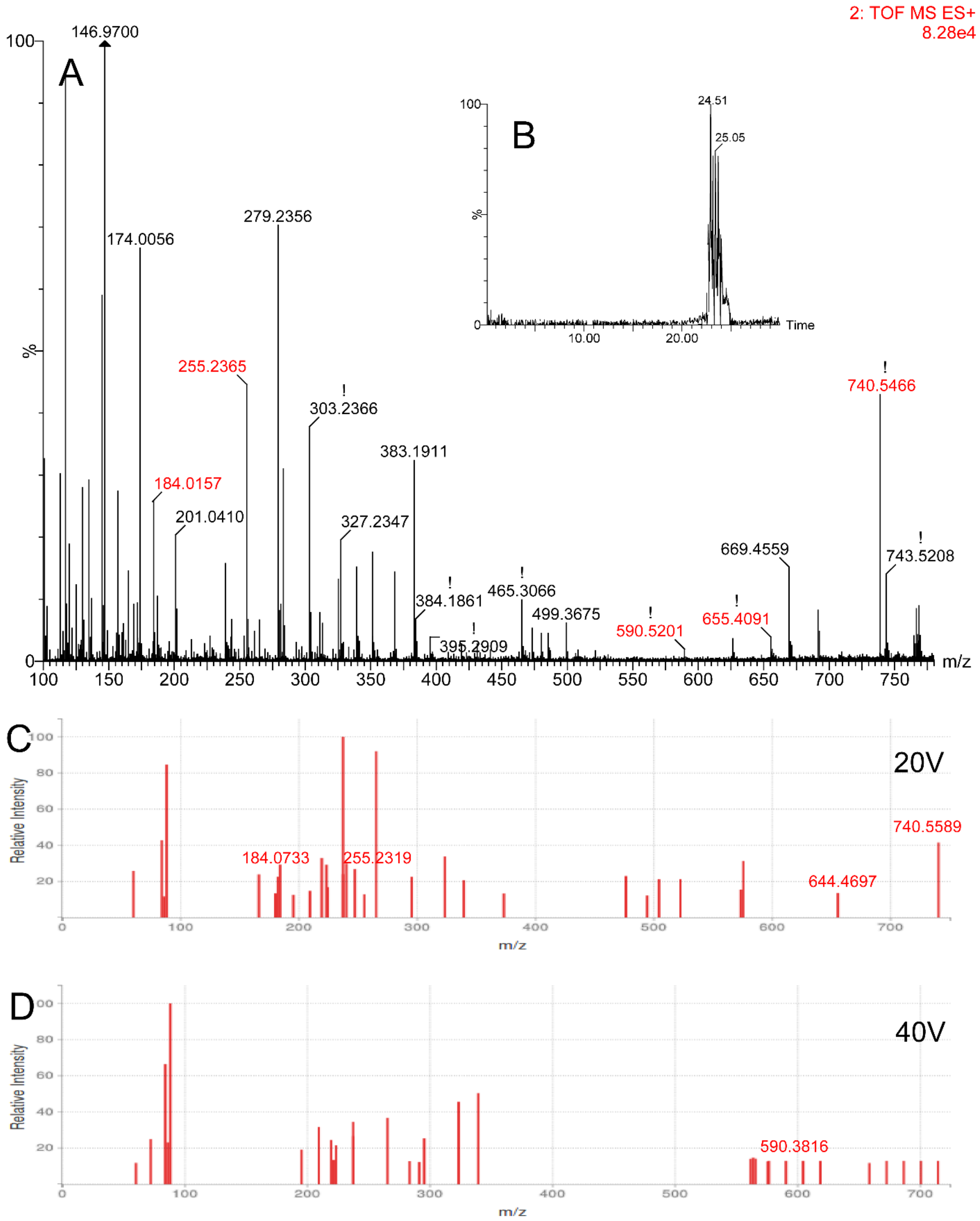

Figure S17. MS/MS spectra of the identified potential metabolic marker **S17** Pc(18:1/16:1) (A) and the chromatographic peak of Pc(18:1/16:1) (B). The reference spectra from HMDB database with in voltage 20V (C) and voltage of 40V (D).

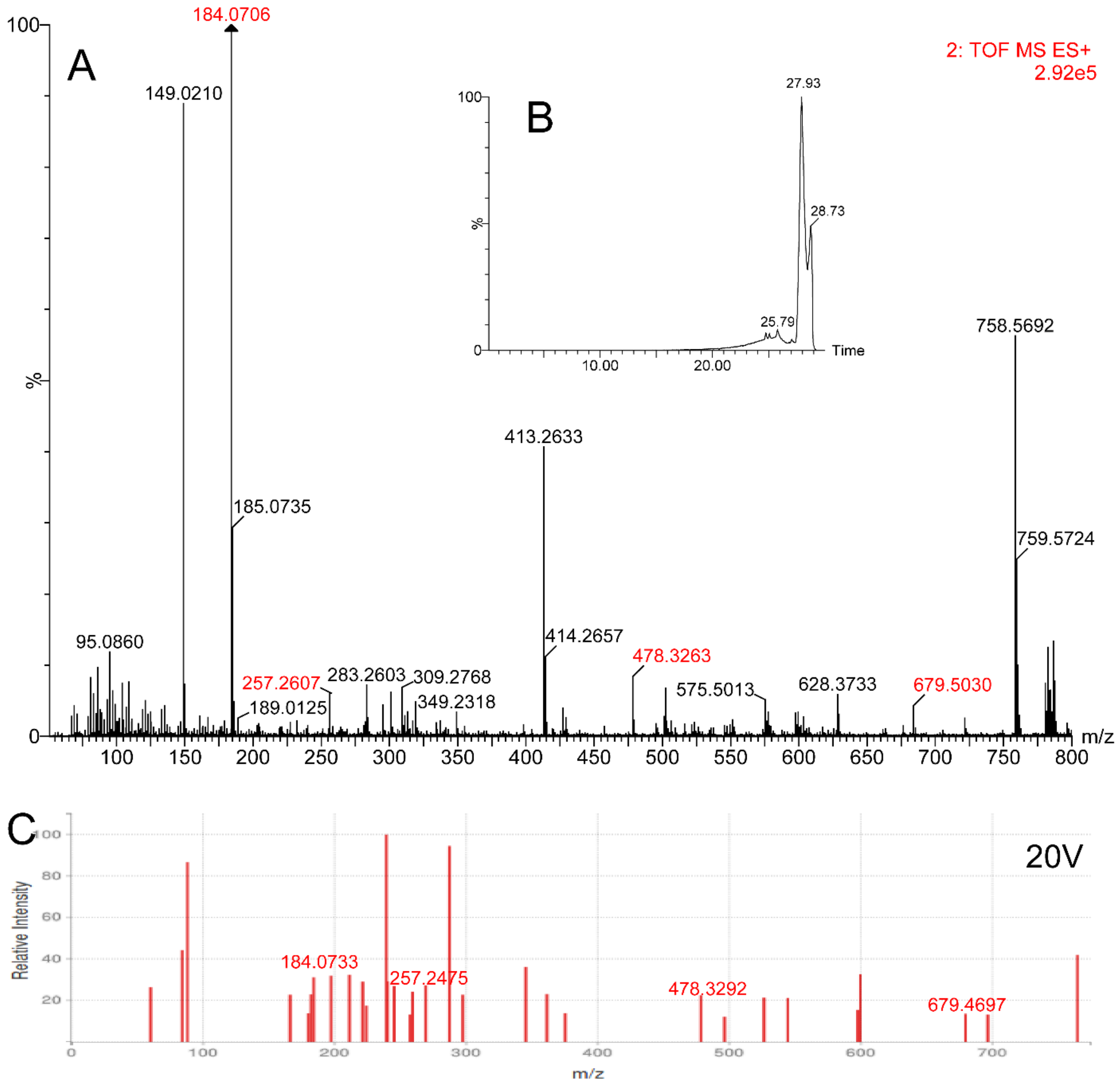

Figure S18. MS/MS spectra of the identified potential metabolic marker **S18** Pc(20:4/16:0) (A) and the chromatographic peak of Pc(20:4/16:0) (B). The reference spectra from HMDB database with in voltage 20V (C).

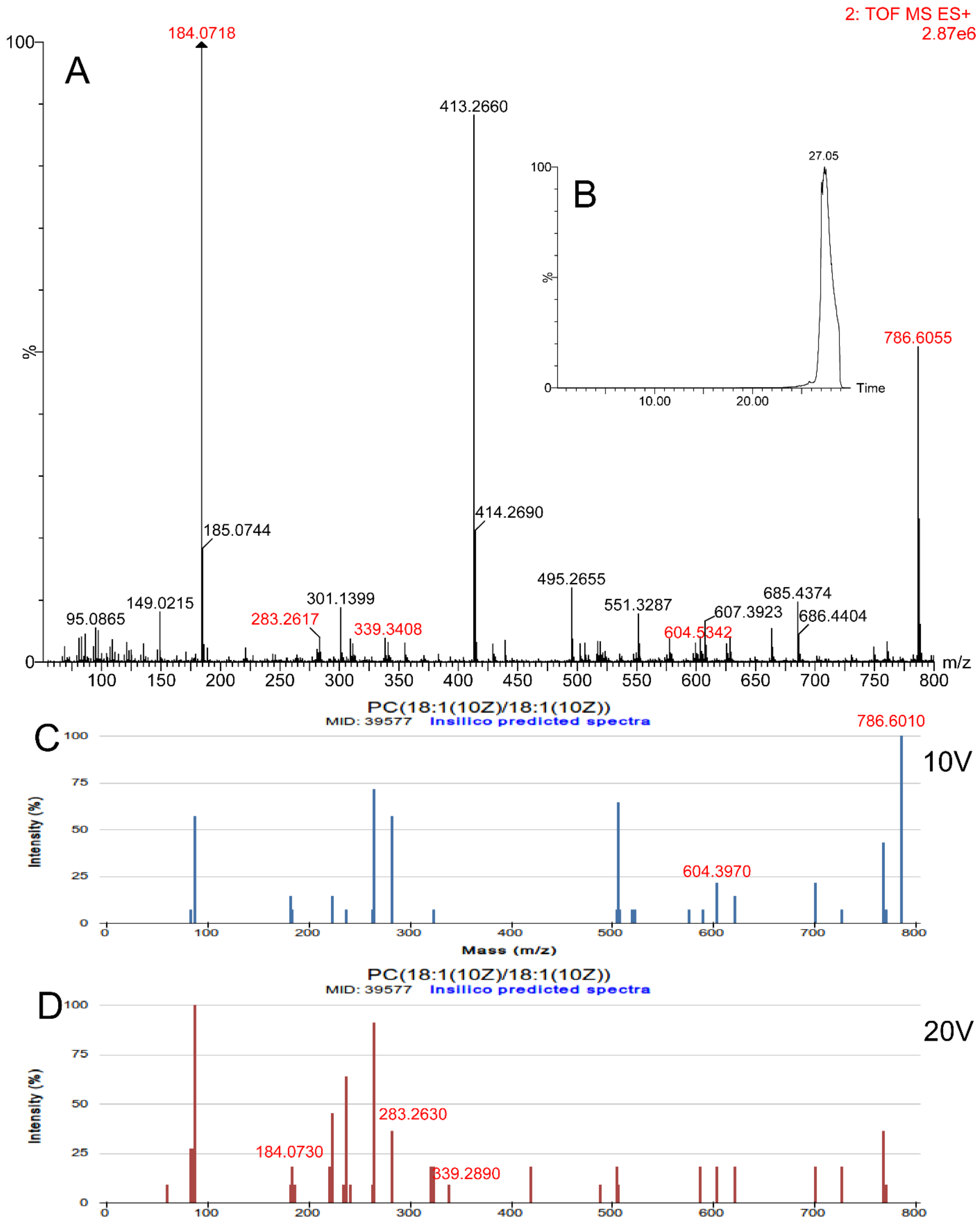

Figure S19. MS/MS spectra of the identified potential metabolic marker **S19** Pc(18:1/18:1) (A) and the chromatographic peak of Pc(18:1/18:1) (B). The reference spectra from METLIN database with in voltage 10V (C) and voltage of 20V (D).

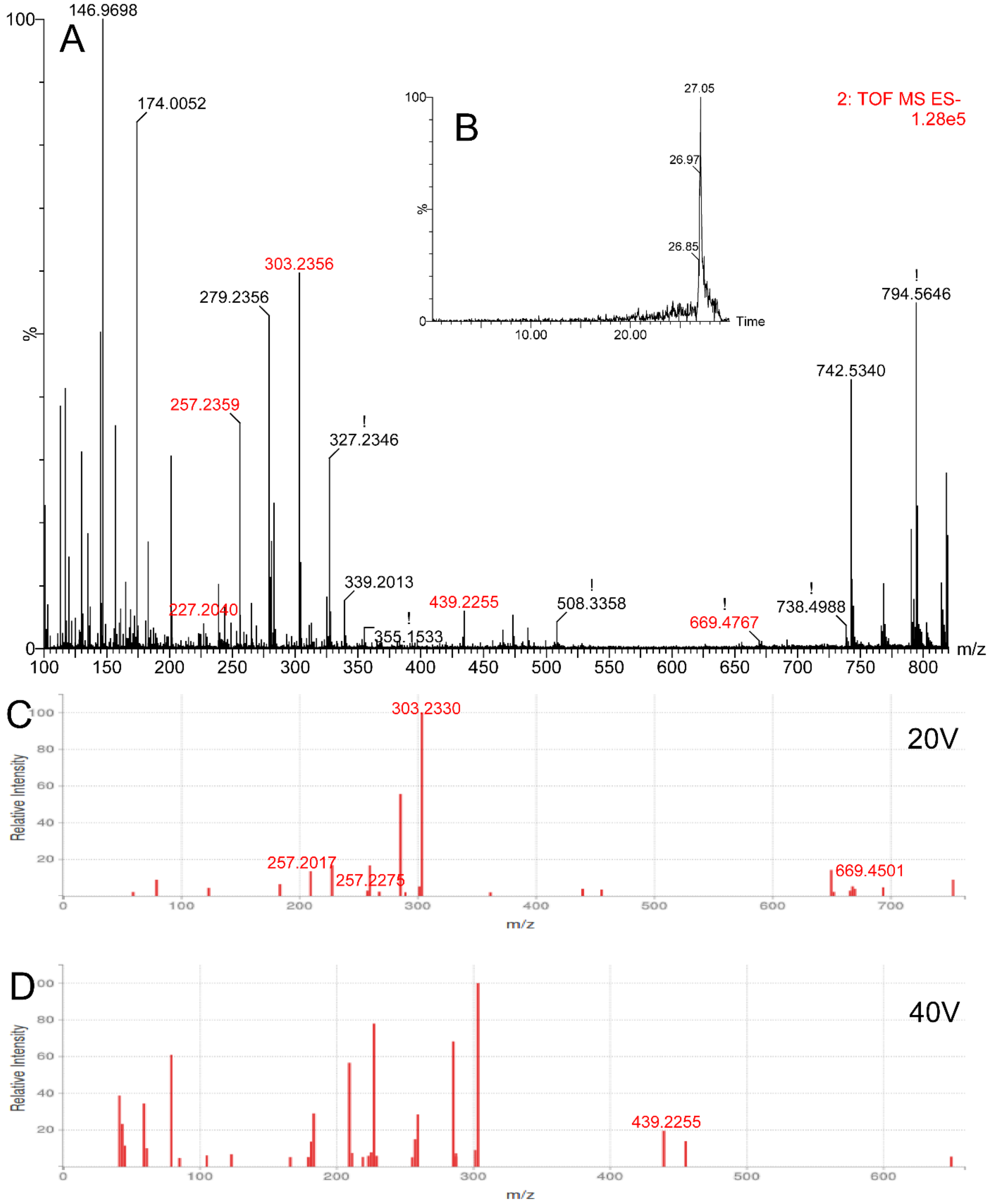

Figure S20. MS/MS spectra of the identified potential metabolic marker **S20** Pc(20:4/14:0) (A) and the chromatographic peak of Pc(20:4/14:0) (B). The reference spectra from HMDB database with in voltage 20V (C) and voltage of 40V (D).

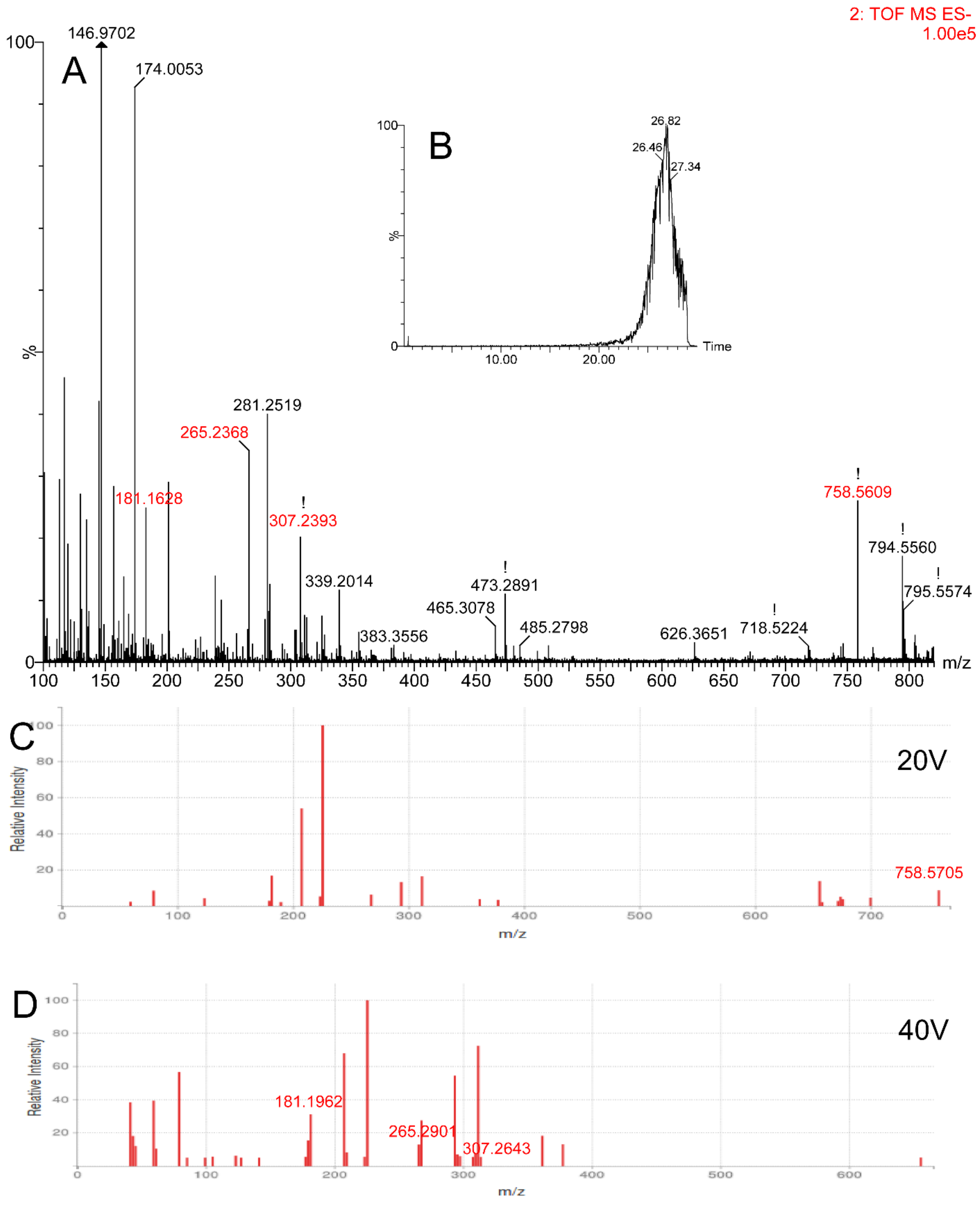

Figure S21. MS/MS spectra of the identified potential metabolic marker **S21** Pc(14:1/20:0) (A) and the chromatographic peak of Pc(14:1/20:0) (B). The reference spectra from HMDB database with in voltage 20V (C) and voltage of 40V (D).

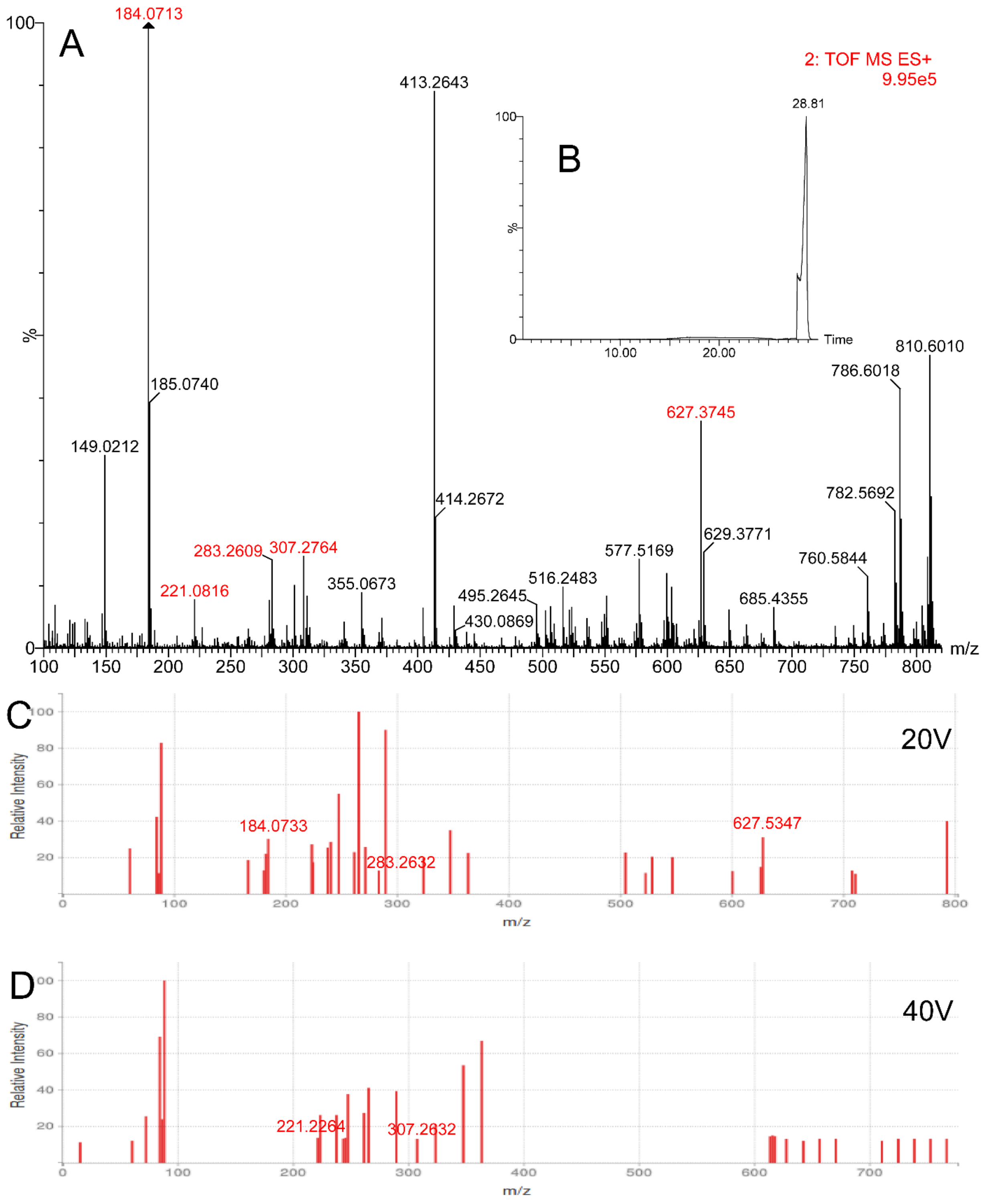

Figure S22. MS/MS spectra of the identified potential metabolic marker **S22** Pc(20:3/18:1) (A) and the chromatographic peak of Pc(20:3/18:1) (B). The reference spectra from HMDB database with in voltage 20V (C) and voltage of 40V (D).

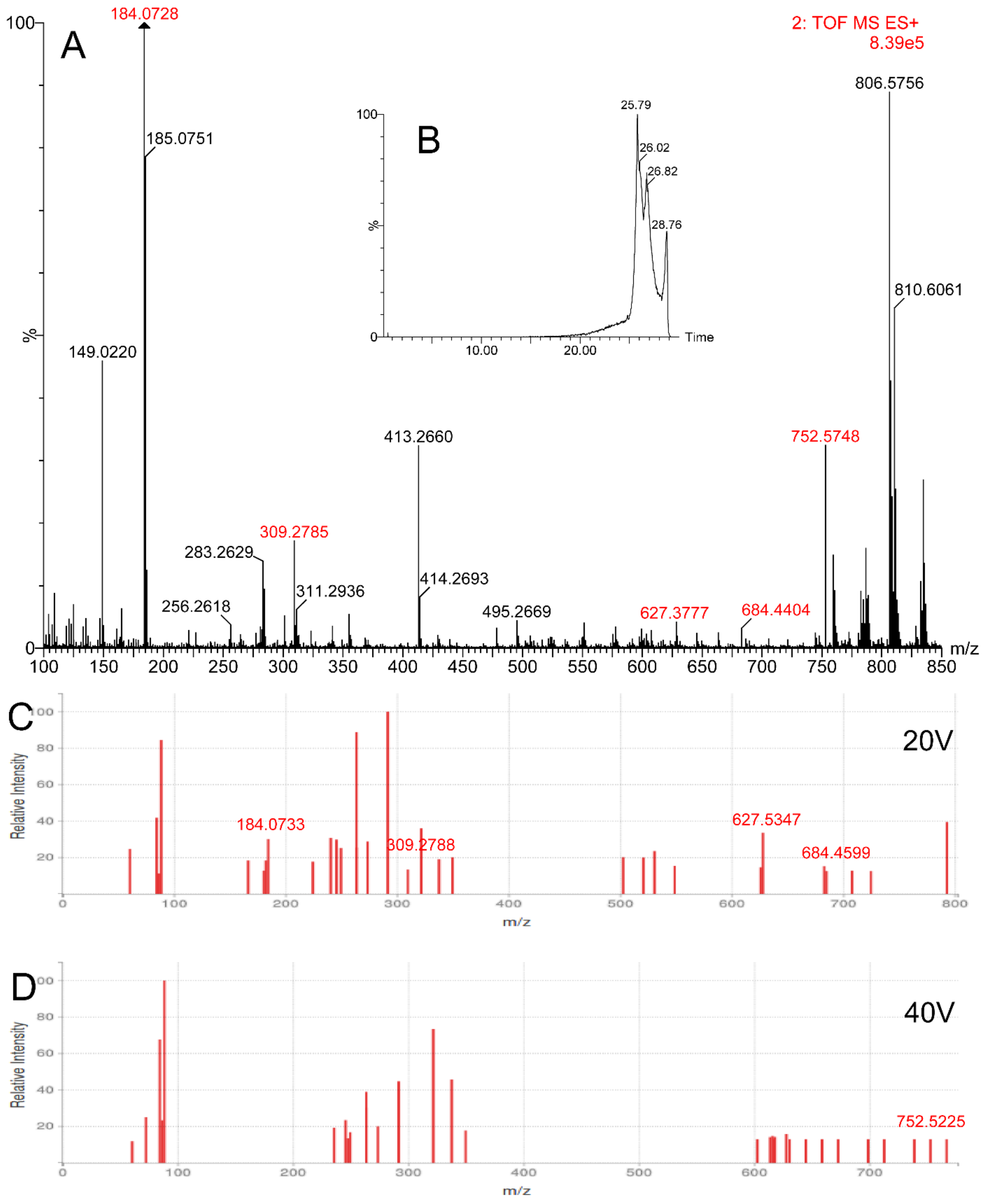

Figure S23. MS/MS spectra of the identified potential metabolic marker **S23** Pc(18:2/20:2) (A) and the chromatographic peak of Pc(18:2/20:2) (B). The reference spectra from HMDB database with in voltage 20V (C) and voltage of 40V (D).

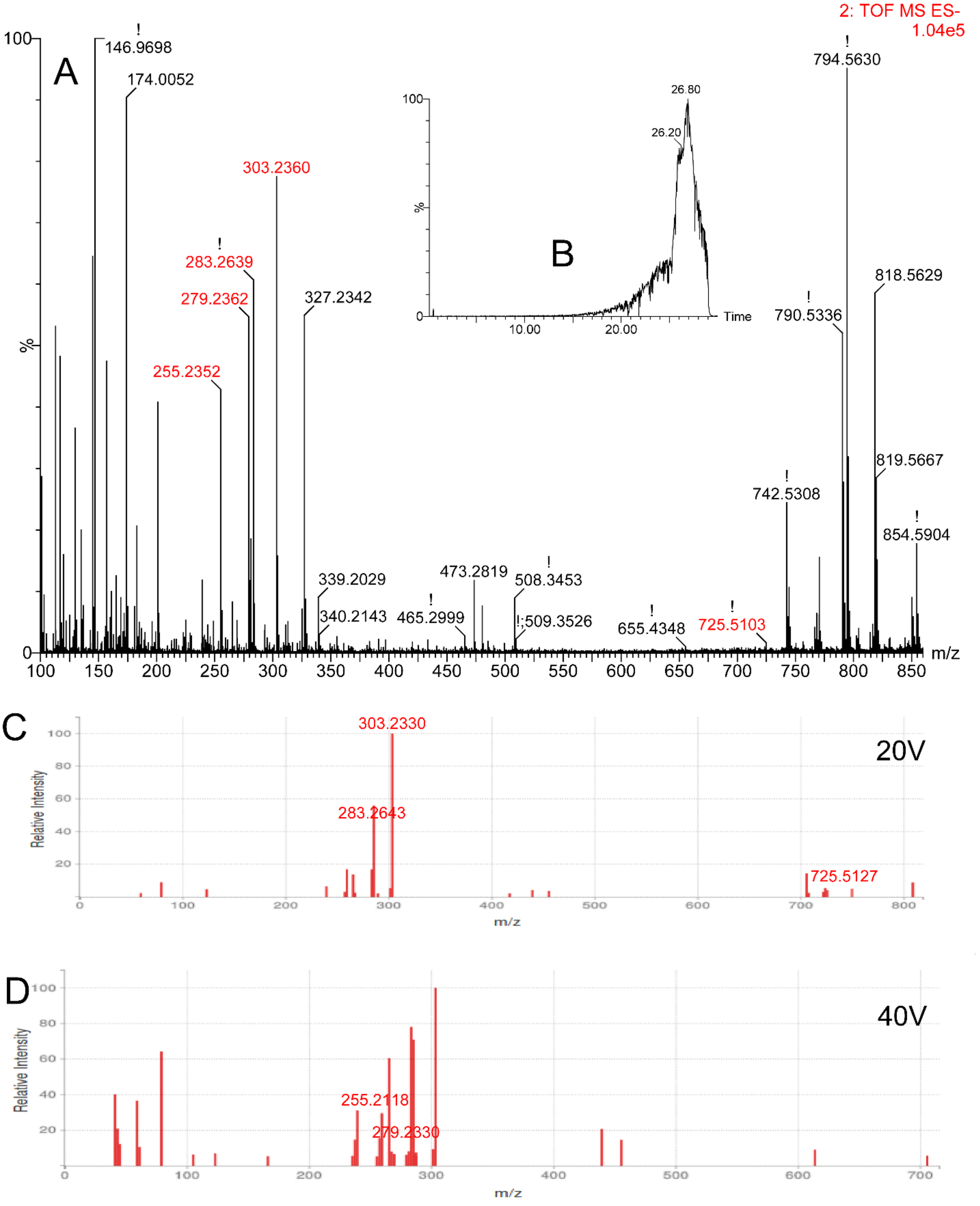

Figure S24. MS/MS spectra of the identified potential metabolic marker **S24** Pc(20:4/18:0) (A) and the chromatographic peak of Pc(20:4/18:0) (B). The reference spectra from HMDB database with in voltage 20V (C) and voltage of 40V (D).

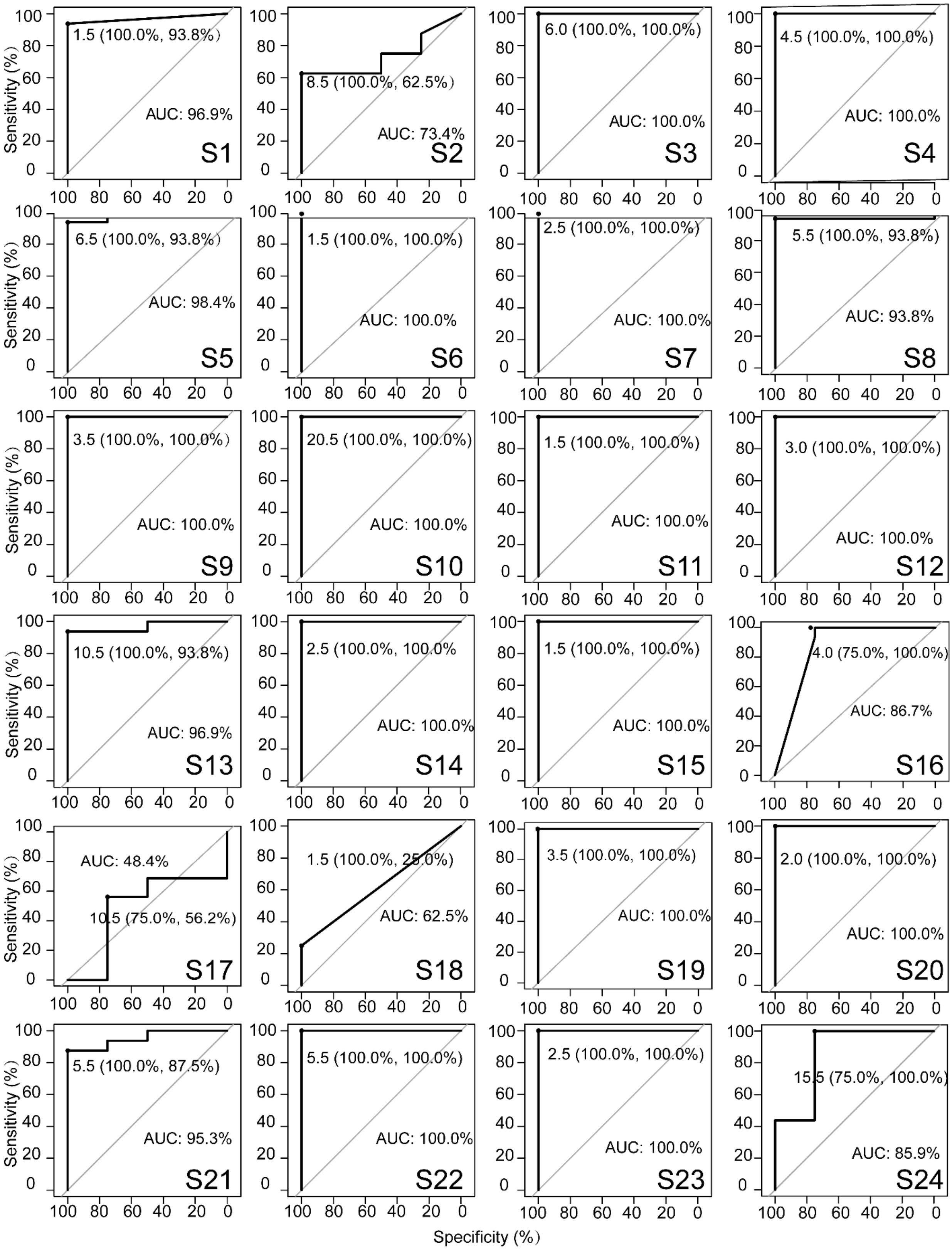

Figure S25. Estimation on the accuracy of all distinct metabolites to be biomarkers. All metabolites but S2, S16, S17, S18, S24 have the potential to be biomarkers with high diagnostic accuracy. AUC, Area Under Curve.
